# Supplementary material for: Allosteric regulation and crystallographic fragment screening of SARS-CoV-2 NSP15 endoribonuclease
Source: Nucleic Acids Res. 2023 Apr 28;51(10):5255–70. doi: 10.1093/nar/gkad314 (PMC10250223; doi:10.1093/nar/gkad314)
Supplement: gkad314_Supplemental_Files [file gkad314_supplemental_files.zip › Supplementary datasets.zip!/139/Refine_1/01-BUSTER/Cycle-2/LIST.html]

Refining

**BUSTER 2.X**: A suite of programs for macromolecular structure refinement by maximum-likelihood and Bayesian methods  
Copyright © 1992-2020 Global Phasing Ltd.  
All rights reserved.  
Authors: G. Bricogne, E. Blanc, M. Brandl, C. Flensburg, P. Keller, W. Paciorek, P. Roversi, O. Smart, C. Vonrhein, T. Womack  

Please reference use of the software as:

- BUSTER-TNT 2.X, Global Phasing Ltd, Sheraton House, Cambridge CB3 0AX, UK

Version: 2.10.3 for linux64-ifort made on May 20 2020 at 01:14:34  
Licenced to: Diamond Light Source, Scientific Software  

```
---


### Parsing of the BUSTER cardfile

TITLE : autoBUSTER refinement

   Cell information

   Symmetry information

 Using CCP4 X-ray form factors.

   Missing structure chemical composition

 Using ATOM block for chemical composition.


   Shell information

### Parsing of the TNT cardfile


---


### Reading observations file

 Number of reflexions in input:  82647

      Number of accepted reflexions:  82309

      Number of rejected reflexions:      0

   Reflexions in test set:            4081

   Reflexions in working set:        78228


---


### 1. Generating initial model

 2.10 * Shannon sampling rate. NX NY NZ:   360  360  240


  Effective Shannon rates are:    2.50    2.50    2.35


   Allocated BUFFER of size:    119.312 Mb.

   Initial Geometry Information


 No missing atoms declared


   Summary of model volumes

 No missing atoms declared: no prior computed


 Computing bulk solvent model scattering


 NCSMASK PDB radius for solvent mask:     2.15 Angstrom

 running PDBSET (setting cell and spacegroup P1) ... done
 running NCSMASK [1] (creating mask, SMOOTH 0.906025) ... done
 running MAPDUMP (to check if reduction to unit cell is needed) ... done
 running MAPMASK (converting mask to map) ... done

   Generation of babslv by theta-filter

   Electron density in frg mask:  0.420 Å-1

 FOBS  put on rough absolute scale by Wilson plot :
           K        =  0.62639E+02
           B        =     -7.282

 

### Cycle 0001: MaxLik scaling and refinement


   MaxLik Scaling Cycle 0001

   Wilson plot

   Statistics in resolution bins just after scaling
```

|  |  |
| --- | --- |
|  | - Rxpct factors plot- Rxpct factors table- <Log-Lik Gain>- Ln Sigma A- Correlation Coefficients |

```
   <Log-Likelihood> of null-hypothesis

Structure Factor Amplitudes


   Average Figure of Merit

 Switching off refinement of overall temperature factor


 Added B =     0.4880 Å2 to all atoms in fragment


 Added B =     0.4880 Å2 to B_SOLV

```
gelly refinement module
 Authors (2004-2009) O. S. Smart, C. Flensburg, W. Paciorek, C. Vonrhein, 
                     M. Brandl, T. O. Womack and G. Bricogne
         (2010-2011) O. S. Smart, C. Flensburg, W. Paciorek, C. Vonrhein, 
                     T. O. Womack and G. Bricogne
         (2012-2014) O. S. Smart, C. Flensburg, W. Paciorek, C. Vonrhein, 
                     and G. Bricogne
         (2015-2020) G. Bricogne, C. Flensburg, W. Paciorek, C. Vonrhein
 Copyright © 2004-2020 Global Phasing Ltd. 
 All rights reserved.
 gelly module compilation date May 20 2020 01:11:20
 
 gelly uses routines from TNT geometry. References: 
     Tronrud, D. E., Ten Eyck, L. F., Matthews, B. W. (1987)
     "An Efficient General-Purpose Least-Squares Refinement Program 
     for Macromolecular Structures". Acta Crystallogr A, 43:489-501.
     Tronrud, D. E. (1997). "The TNT Refinement Package", 
     in Macromolecular Crystallography, Part B, Eds Charlie Carter 
     and Robert Sweet, Methods in Enzymology, 277:306-319.
 wildcard matching function by Julian Robichaux http://www.nsftools.com

 No -random_seed flag found. Random number stream initialised using:
2147483562
2147483398

 Control flags set to:  -autoncs -hydrogens_input_occ2zero \
                     -hydrogens_output_occ2heavy -screen \
                     100 -screen_sigma \
                     3.0 -keeppdb \
                     1000000 -taomonitor_verbose \
                     2 
 

 ############### Starting setup via TNT routines

 Setting up tnt routines including license check:

                   TNT Stereochemistry Program
 Version: 5.22.3.0
 Licence for Diamond Light Source, Scientific Software is valid
 
 Using TNT geometry routines to load the tnt control file: gelly.tnt
 Using TNT routines to load initial coordinates from file fragment.cor
 Expecting 6119 atoms

 Found     2192 "NOTE BUSTER_RESIDUE_ATOM_DATA" cards
 Have copied information for    6119 atoms from TNT to gelly's store 
 
 The TNT WEIGHT cards used by this run:
 -----WEIGHT       BOND     2.0
 -----WEIGHT       ANGLE    2.0
 -----WEIGHT       IMPROPER 0.0
 -----WEIGHT       TORSION  2.0
 -----WEIGHT       PSEUDO   0.0
 -----WEIGHT       TRIGONAL 2.0
 -----WEIGHT       PLANE    5.0
 -----WEIGHT       CONTACT  5.0
 -----WEIGHT       BCORREL  10.0
 -----WEIGHT       CHIRAL   5.0
 -----WEIGHT       NCS      50.0
 -----WEIGHT       IDEAL    4.0
 -----WEIGHT       RFACTOR  2.21
 
 Dictionary versions picked up from NOTE BUSTER_RESTRAINT_DICT cards as: 
 ------ protgeo_eh99.dat (V1.6.4.17) 20191001  STANDARD AMINO ACID DICTIONARY. BONDS AND ANGLES FROM ENGH AND HUBER EH99. OTHER VALUES BASED ON PREVIOUS TNT OR TAKEN FROM CCP4. INCLUDES HYDROGEN ATOMS.
 ------ exoticaa.dat (V1.3.4.15) 20200327  COLLECTION OF NON-STANDARD AMINO ACIDS, MAINLY EH91 WITHOUT IDEAL DISTANCE INFO
 ------ nuclgeo.dat (V1.13.4.8) 20190909
 ------ bcorrel.dat (V1.15.10.2) 20190616
 ------ contact.dat (V1.15.12.10) 20191121
 ------ idealdist_contact.dat (V1.3.4.12) 20191001  IDEAL-DISTANCE CONTACT TERM DATA AS USED IN PROLSQ. VALUES USED HERE ARE BASED ON DATA HARVESTING OF PDB AS OF 20190109.
 ------ restraints for LIG (Unknown) from cif dictionary Z85934875.cif
 ------ restraints for CIT (CITRIC ACID) from cif dictionary CIT.cif; generated by GRADE_PDB_LIGAND 1.2.12 (October 4 2016) using MOGUL 1.7.2(RC1), CSD as538be, with quantum mechanics RM1 ((DatabaseID PDB-CIT))
 ------ assume.dat (V1.9.4.2) 20180705
 
 Have picked up spacegroup name for pdb output as 'P 63      '
 Setting up Chain/Residue arrays
 
 Have command line specification -autoncs*
     Have found       1 NCS relations
     Pairwise matching residue number and type between chains: 
                    A        B        S    
          A         .       99%       -   
          B                  .        -   
          S                           .   
       Table key--                                                       
          all   : two chains have 100% match and same number of residues 
         multi  : ditto and they contribute to a multi chain ncs cluster 
          ***%  : two chains have >80% matching residue number/type      
           -    : chains appear to be unrelated                          
      chain A     have identified ncs to     1 other chains. So will weight each autoncs by (       6.666/#)=      6.6660
      chain B     have identified ncs to     1 other chains. So will weight each autoncs by (       6.666/#)=      6.6660
      chain S     have identified ncs to     0 other chains. 
     For .Gelly cards produced see below - after pruning
 
 Setting up BUSTER_SET groups
 Have found    89 BUSTER_SET cards
 Now silently applying automatic/dictionary set definitions. For a verbose listing use '-verbose_set' flag.
 Applying user defined set definitions:
 =====NOTE BUSTER_SET ncsautoXcld = Water --> set 'ncsautoXcld' now has   581 atoms 
 =====NOTE BUSTER_SET ncsautoABset = Chain_B \ ncsautoXcld --> set 'ncsautoABset' now has  2770 atoms 
 Have defined    48 sets: 
      set:                 'All' has   6119 atoms in   1281 residues - automatically defined
      set:               'Empty' has      0 atoms in      0 residues - automatically defined
      set:            'ExoticAA' has      0 atoms in      0 residues - defined by dictionary file
      set:           'ExoticNUC' has      0 atoms in      0 residues - defined by dictionary file
      set:              'NotHET' has   5514 atoms in    698 residues - defined by dictionary file
      set:             'Chain_A' has   2768 atoms in    350 residues - automatically defined
      set:             'Chain_B' has   2770 atoms in    350 residues - automatically defined
      set:             'Chain_S' has    581 atoms in    581 residues - automatically defined
      set:               'Water' has    581 atoms in    581 residues - defined by dictionary file
      set:          'StdProtein' has   5514 atoms in    698 residues - defined by dictionary file
      set:             'Protein' has   5514 atoms in    698 residues - defined by dictionary file
      set:                'Back' has   2794 atoms in    698 residues - defined by dictionary file
      set:                'Side' has   2720 atoms in    650 residues - defined by dictionary file
      set:        'RnaCanonical' has      0 atoms in      0 residues - defined by dictionary file
      set:        'DnaCanonical' has      0 atoms in      0 residues - defined by dictionary file
      set:     'RnaDnaCanonical' has      0 atoms in      0 residues - defined by dictionary file
      set:     'DnaRnaCanonical' has      0 atoms in      0 residues - defined by dictionary file
      set:         'RnaExtended' has      0 atoms in      0 residues - defined by dictionary file
      set:         'DnaExtended' has      0 atoms in      0 residues - defined by dictionary file
      set:      'RnaDnaExtended' has      0 atoms in      0 residues - defined by dictionary file
      set:      'DnaRnaExtended' has      0 atoms in      0 residues - defined by dictionary file
      set:                 'Dna' has      0 atoms in      0 residues - defined by dictionary file
      set:                 'Rna' has      0 atoms in      0 residues - defined by dictionary file
      set:              'RnaDna' has      0 atoms in      0 residues - defined by dictionary file
      set:              'DnaRna' has      0 atoms in      0 residues - defined by dictionary file
      set:             'Nucleic' has      0 atoms in      0 residues - defined by dictionary file
      set:             'Polymer' has   5514 atoms in    698 residues - defined by dictionary file
      set: 'BufferWeichenberger' has     13 atoms in      1 residues - defined by dictionary file
      set:    'BufferAdditional' has      0 atoms in      0 residues - defined by dictionary file
      set:              'Buffer' has     13 atoms in      1 residues - defined by dictionary file
      set:         'MetalAlkali' has      0 atoms in      0 residues - defined by dictionary file
      set:  'MetalAlkalineEarth' has      0 atoms in      0 residues - defined by dictionary file
      set:     'MetalTransition' has      0 atoms in      0 residues - defined by dictionary file
      set: 'MetalPostTransition' has      0 atoms in      0 residues - defined by dictionary file
      set:    'MetalLanthanides' has      0 atoms in      0 residues - defined by dictionary file
      set:               'Metal' has      0 atoms in      0 residues - defined by dictionary file
      set:         'SugarAlphaD' has      0 atoms in      0 residues - defined by dictionary file
      set:         'SugarAlphaL' has      0 atoms in      0 residues - defined by dictionary file
      set:               'Sugar' has      0 atoms in      0 residues - defined by dictionary file
      set:               'Other' has     24 atoms in      2 residues - defined by dictionary file
      set:            'Hydrogen' has      0 atoms in      0 residues - defined by dictionary file
      set:         'nonHydrogen' has   6119 atoms in   1281 residues - defined by dictionary file
      set:             'OccZero' has      0 atoms in      0 residues - defined by dictionary file
      set:          'OccNonZero' has   6119 atoms in   1281 residues - defined by dictionary file
      set:             'OccFull' has   6076 atoms in   1278 residues - defined by dictionary file
      set:          'OccNonFull' has     43 atoms in      3 residues - defined by dictionary file
      set:         'ncsautoXcld' has    581 atoms in    581 residues - user specified
      set:        'ncsautoABset' has   2770 atoms in    350 residues - user specified
 No command line arguments '-occ_allhet' or '-occ_restyp' so will not setup automated occcupancy refinement
 
 Interpreting CONSTANT and FREE cards: 
 =====CONSTANT     OCC
 Have found and interpreted      1 CONSTANT cards in the TNT input. 
 Before any COMBINE - Number of atoms with fixed XYZ =     0
 Before any COMBINE - Number of atoms with fixed B   =     0
 Before any COMBINE - Number of atoms with fixed OCC =  6119
 Before any COMBINE - Number of atoms with fixed BIJ =   581
                            cf total number of atoms =  6119
 
 There are no COMBINE cards to be found in the TNT card input 
 
 There are no NOTE BUSTER_COMBINE_RES cards to be found in the TNT card input 

 ############### Working out full connectivity list


 ############### Working out equivalent atoms


 Residue with most atoms given below:
        A|58:N(TRP)         
        A|58:CA(TRP)        
        A|58:CB(TRP)        
        A|58:CG(TRP)        
        A|58:CD1(TRP)       
        A|58:NE1(TRP)       
        A|58:CE2(TRP)       
        A|58:CD2(TRP)       
        A|58:CE3(TRP)       
        A|58:CZ3(TRP)       
        A|58:CH2(TRP)       
        A|58:CZ2(TRP)       
        A|58:C(TRP)         
        A|58:O(TRP)         


 Have no '-equiv_check_plus' argument - so will take atom type into
 account when checking for equivalence of otherwise identical atoms.
 

 ############### Looking at dihedrals


 ############### Looking at chirals

 NOTE BUSTER_CHIRAL_ANGLECUT card found - read value=    -31.00 degs from it
 NOTE BUSTER_CHIRAL_SIGMA card found - read value=      1.70 degs from it
 RANGE allowed for B variables in refinement:   from      3.00000 to    300.00000

 ############### Preparing for PDB output


 ############### Processing extra restraints explicitly defined

 No 'NOTE BUSTER_TARGET' cards or -target?? args found (used to read coordinates files for soft ncs to external)
 No 'NOTE BUSTER_OCCSUM'     cards found - so not using sum of occupancy restraints
 No 'NOTE BUSTER_DISTANCE'   cards found - so not setting up any additional atom-atom distance restraints
 No 'NOTE BUSTER_UTILANGLE'  cards found - so not setting up any additional restraints on any angle
 No 'NOTE BUSTER_UTILTOR'    cards found - so not setting up any additional sinusoidal restraints on torsions
 
 --- Have some similarity definitions 
     Similarity restraints on RMSD use superposition routines from: 
     Coutsias, E.A., Seok, C., Dill, K.A.(2004)
     "Using quaternions to calculate RMSD", J. Comput. Chem., 25:1849-1857 

 Have found    1 'NOTE BUSTER_SIM_DEFINE' cards 
     Maximum number of chains involved in any SIM group (excluding the template)=     1


 ########## Interpreting card: NOTE BUSTER_SIM_DEFINE ncsautoAB ncsautoABset A
     Number of atoms in template set=  2770
     Number of related chains (other than template) in SIM set=    1
     List of chains to be restrained (excluding template)=   A   
     Pairwise RMSD, XYZ in Angstroms, for SIM group: ncsautoAB
                 B         A   
     B         -----     ----- 
     A         0.726     ----- 
     Overall RMSD,XYZ for the SIM group is     0.726 Angstroms
     Pairwise rms deviation of [individual B's minus their set/chain average] in Angs**2
                 B         A         Bavg
     B         -----     -----     57.11
     A         49.65     -----     91.83
     Overall RMSD,B   for the SIM group is    49.649 Angs**2
     The number of common atoms for the above fits:
                 B         A   
     B         -----     ----- 
     A          2757     ----- 
     (compared to the template set that has    2770 atoms in it)
     List of atoms that are absent in one or more chains for this group: 
                                    B    A   
                    C1      401     +    -
                    O1      401     +    -
                    O2      401     +    -
                    C2      401     +    -
                    C3      401     +    -
                    O7      401     +    -
                    C4      401     +    -
                    C5      401     +    -
                    O3      401     +    -
                    O4      401     +    -
                    C6      401     +    -
                    O5      401     +    -
                    O6      401     +    -
     (key  +  atom present for this chain 
           -  atom absent  for this chain)
 transforms for each of the superpositions orthogonal matrix r11 r12 r13 r21 r22 r23 r31 r32 r33 tx ty tz (PDBSET syntax): 
TRANS_BA -0.873718 -0.485659  0.027413 -0.485806  0.874065  0.001449 -0.024665 -0.012052 -0.999623    45.254   -12.569    15.291
 reverse transforms 
TRANS_AB -0.873718 -0.485806 -0.024665 -0.485659  0.874065 -0.012052  0.027413  0.001449 -0.999623    33.810    33.148    14.063
 
 Setting up LSSR interatomic distance restraints
 Interpreting card: NOTE BUSTER_SIM_RESTRAIN_LSSR ncsautoAB 6.666
        setting up interatomic distance pair restraints with:
        Dmin=         0.00
        Dmax=         5.50
        sigma=        0.20
        Vmax=         3.00
        weight=       6.67
        number of distance pairs defined for this set is      35700
 Have not supplied either '-sim_swap_equiv' or '-sim_swap_equiv_plus' argument 
   so will not try swaping equivalent atoms to improve NCS and/or target agreement.
 
 NCS by LSSR outlier analysis
 -autoncs option automatic NCS prune routine -------- 
 Residues pruned out by gradient condition:
       
 Residues pruned out by function/max condition:
          A|149     A|150     A|173     A|187     A|188     A|201     A|215 
          A|216     A|220     A|226     A|259     A|264     A|307     A|345 
         A|ENDA     B|149     B|150     B|173     B|187     B|188     B|201 
          B|215     B|216     B|220     B|226     B|259     B|264     B|307 
          B|345    B|ENDA 
  
 Total number of residues for which LSSR restraints have been pruned out is       30 out of      698 participating
 Number of LSSR restraints pruned out is      2136 this is    6.0% of the total           35700
  
 -autoncs .Gelly cards suitable for adaptation for manual definition/pruning
           (note auto pruning is done in a slightly different way so exact results may differ slightly)
 -autoncs option .Gelly cards also written to file  auto_gelly_cards.txt
NOTE BUSTER_SET ncsautoXcld = Water    # card added - produced by -autoncs
NOTE BUSTER_SET ncsautoXcld = ncsautoXcld + { B|149 B|150 B|173 B|187 B|188 }    # card added - produced by -autoncs
NOTE BUSTER_SET ncsautoXcld = ncsautoXcld + { B|201 B|215 B|216 B|220 B|226 }    # card added - produced by -autoncs
NOTE BUSTER_SET ncsautoXcld = ncsautoXcld + { B|259 B|264 B|307 B|345 B|ENDA }    # card added - produced by -autoncs
NOTE BUSTER_SET ncsautoABset = Chain_B \ ncsautoXcld    # card added - produced by -autoncs
NOTE BUSTER_SIM_DEFINE ncsautoAB ncsautoABset A    # card added - produced by -autoncs
NOTE BUSTER_SIM_RESTRAIN_LSSR ncsautoAB 6.666    # card added - produced by -autoncs

 LSSR function contribution before pruning    154619.3
                             after pruning    131579.4
 Graph of which residues pruned from LSSR: ncs_pruned.mtv
 Graph of LSSR function outliers ncs_function_outliers.mtv
 Graph of LSSR gradient outliers ncs_gradient_outliers.mtv
 LSSR - analysis of distance difference distribution for NCS
         DeltaD less than 0.5 Angs:  proportion of population= 96.01% average DeltaD of subset     0.114 Angs
 Histogram of LSSR deltaD distribution ncs_histogram.mtv
 
 Interpreting card: NOTE BUSTER_TLS_SET tls1 { A|* }
 Interpreting card: NOTE BUSTER_TLS_SET tls2 { B|* }
 Interpreting card: NOTE BUSTER_TLS_SET tls1 { A|* }
 Interpreting card: NOTE BUSTER_TLS_T   tls1     -4.99974E-02  8.17891E-02  2.87735E-02 -4.37364E-02  3.25931E-02 -5.24938E-02
 Interpreting card: NOTE BUSTER_TLS_L   tls1      1.34630E+00  5.19551E-01  5.36731E-01 -3.76866E-01 -6.37599E-01  1.69109E-01
 Interpreting card: NOTE BUSTER_TLS_S   tls1      1.07403E-01 -2.67331E-01  3.34085E-01 -2.69031E-01  1.27949E-01 -7.05510E-02  3.27382E-02 -1.30945E-01
 Interpreting card: NOTE BUSTER_TLS_O   tls1     -2.32263E+01 -8.41675E+01  1.09752E+01
 Interpreting card: NOTE BUSTER_TLS_SET tls2 { B|* }
 Interpreting card: NOTE BUSTER_TLS_T   tls2     -6.98594E-02 -1.86037E-02  3.61751E-02  9.32305E-03  3.30742E-02 -3.60360E-03
 Interpreting card: NOTE BUSTER_TLS_L   tls2      6.97425E-01  3.84731E-01  4.14949E-01  2.96503E-01 -4.03999E-01 -3.04761E-01
 Interpreting card: NOTE BUSTER_TLS_S   tls2      8.24345E-03  4.73999E-02 -7.39498E-02  8.97429E-02  1.02197E-01  9.18372E-03 -3.17101E-02  3.38103E-02
 Interpreting card: NOTE BUSTER_TLS_O   tls2      2.22079E+01 -7.23190E+01  3.56420E+00
 Interpreting card: NOTE BUSTER_TLS_FIX tls1 ALL
 Interpreting card: NOTE BUSTER_TLS_FIX tls2 ALL


 TLS information. Total number of groups:     2


 Group   Natoms    FixRB   FixTLS   Group Tag    Group Spec
     1     2768      Y        Y     tls1    { A|* }
     2     2770      Y        Y     tls2    { B|* }
 
NOTE BUSTER_TLS_SET tls1 { A|* }
NOTE BUSTER_TLS_T   tls1     -4.99974E-02  8.17891E-02  2.87735E-02 -4.37364E-02  3.25931E-02 -5.24938E-02
NOTE BUSTER_TLS_L   tls1      1.34630E+00  5.19551E-01  5.36731E-01 -3.76866E-01 -6.37599E-01  1.69109E-01
NOTE BUSTER_TLS_S   tls1      1.07403E-01 -2.67331E-01  3.34085E-01 -2.69031E-01  1.27949E-01 -7.05510E-02  3.27382E-02 -1.30945E-01
NOTE BUSTER_TLS_O   tls1     -2.32263E+01 -8.41675E+01  1.09752E+01
NOTE BUSTER_TLS_FIX tls1 ALL
 
NOTE BUSTER_TLS_SET tls2 { B|* }
NOTE BUSTER_TLS_T   tls2     -6.98594E-02 -1.86037E-02  3.61751E-02  9.32305E-03  3.30742E-02 -3.60360E-03
NOTE BUSTER_TLS_L   tls2      6.97425E-01  3.84731E-01  4.14949E-01  2.96503E-01 -4.03999E-01 -3.04761E-01
NOTE BUSTER_TLS_S   tls2      8.24345E-03  4.73999E-02 -7.39498E-02  8.97429E-02  1.02197E-01  9.18372E-03 -3.17101E-02  3.38103E-02
NOTE BUSTER_TLS_O   tls2      2.22079E+01 -7.23190E+01  3.56420E+00
NOTE BUSTER_TLS_FIX tls2 ALL


 TLS: mmCIF group specification begin


#
loop_
_pdbx_refine_tls_group.id
_pdbx_refine_tls_group.refine_tls_id
_pdbx_refine_tls_group.beg_PDB_ins_code
_pdbx_refine_tls_group.beg_auth_asym_id
_pdbx_refine_tls_group.beg_auth_seq_id
_pdbx_refine_tls_group.end_PDB_ins_code
_pdbx_refine_tls_group.end_auth_asym_id
_pdbx_refine_tls_group.end_auth_seq_id
_pdbx_refine_tls_group.selection
_pdbx_refine_tls_group.selection_details
   1   1 . A   -1 . A  346 . '{ A|* }'
   2   1 . A  501 . A  501 . '{ A|* }'
   3   2 . B   -1 . B  346 . '{ B|* }'
   4   2 . B  401 . B  401 . '{ B|* }'
#


 TLS: mmCIF group specification end


 Interpreting card: NOTE BUSTER_ANISO_SET Empty
 Interpreting card: NOTE BUSTER_ANISO_WEIGHT SCA  16.0
 Interpreting card: NOTE BUSTER_ANISO_WEIGHT SPH   0.6    0.6
 Interpreting card: NOTE BUSTER_ANISO_WEIGHT SIM   7.8    4.8    2.6    0
 Interpreting card: NOTE BUSTER_ANISO_WEIGHT RIG  21.5   21.5    3.7    0


 After TLS and ANISO: Number of atoms with fixed/combined xyz =     0
 After TLS and ANISO: Number of atoms with fixed/combined B   =     0
 After TLS and ANISO: Number of atoms with fixed/combined OCC =  6119
 After TLS and ANISO: Number of atoms with fixed/combined BIJ =  6119
 After TLS and ANISO: Number of atoms in a TLS group          =  5538
                                     cf total number of atoms =  6119
 

 ############### Setup state vector

 No free_vect_ulim_xyz argument found: using default of  100.000
 No free_vect_llim_xyz argument found: using default of -100.000
 Number of parameters added for TLS description                 =       0
 Number of parameters to describe molecular conformation        =   24476

 ############### Setup free vector

 Number of free parameters to be adjusted in refinement   =   24476

 ############### Setting up own geometry


 No command line argument '-tnt_fn' so will use gelly to do geometry function calculations

 ============= Call TNT functions


 ============= Get TNT symmetry information

 
      Getting symmetry operators from TNT. 
          gelly will classify symmetry using pdb-like convention: 
               SYMOP   SYMMETRY
              NNNMMM   OPERATOR
                1555   X,Y,Z
                2555   -Y,X-Y,Z
                3555   -X+Y,-X,Z
                4555   -X,-Y,1/2+Z
                5555   Y,-X+Y,1/2+Z
                6555   X-Y,X,1/2+Z
          where NNN -> operator number and MMM -> translation vector
 

 ============= Own Gelly geometry/restraints setup


 ----------- Identify bond-types


    Setting up GEOMBONDREC_TYPE_HASH* to be able to map a given bond type (e.g.
    "single") to an internal integer (GBOND_TYPE=1) etc.

    Using default  GEOMBONDREC_TYPE_HASH_STR='SING* 1 DOUB* 2 TRIP* 3 AROM* 4 DELO* 4 METAL* 5':
    to override default use a NOTE BUSTER_GEOMBONDREC_TYPE_HASH_STR gelly card.

 ----------- Classify torsion into peptide omega and other


 ----------- Setup of non-bonded terms

 Ideal-distance contact term setup:
    Restraint function constants (normally these values are from NOTE BUSTER_IDEAL_CONTACT_* cards)
    Sigma for general contacts      0.200 Angs
    Sigma for 1-4 contacts          0.100 Angs (contacts across a torsion)
    Sigma for 1-5 contacts          0.150 Angs
    Sigma for 1-N contacts          0.200 Angs
    Sigma for HBOND pairs           0.100 Angs (contacts between HB_types A and D or B's)
    Contact distance increment for each atom in HBOND          -0.150 Angs 
    Contact distance increment for H-bond acceptor              0.100 Angs 
    Contact distance increment for 1-4 with an O atom          -0.050 Angs 
    Contact distance increment for 1-4 with an N atom          -0.180 Angs 
    Contact distance increment for 1-4 with an C atom          -0.370 Angs 
    Contact distance increment for 1-4 with an S atom          -0.150 Angs 
    Contact distance increment for 1-4 with an P atom          -0.150 Angs 
    Contact distance increment for 1-4 with any other atom     -0.150 Angs 
    All atoms matched for CCP4 atom type.
    Number of CCP4 atom types used is         24 (H-bound and not H-bound counted separately)

 Setting up short/ideal-distance contacts
   Using gelly special position handling. (For TNT instead rerun with argument '-special_tnt')

   No command line argument '-special_all' specified. We will auto-generate
   EXCLUDE cards only for atom-atom contacts across symmetry (i.e. on special
   position) when this involves a contact within a residue to itself.

   Have set up non-bonded exclusions - 1-3 contacts
   Atom A|27:CA         makes the most 1-3 contacts:  6

   Have set up non-bonded exclusions - 1-4 contacts
   Atom A|27:N          makes the most 1-4 contacts:  7

   Have set up non-bonded exclusions - 1-5 contacts
   Atom A|28:C.A        makes the most 1-5 contacts: 10

 ############################################################################################################
 ###################################################### setting up contact terms
 ############################################################################################################


 ============= Call Gelly versions of TNT functions

 WARNING: 1-4 contact without 1-(2,3)-4 data for A|501:C01(LIG)       and A|501:O04(LIG)       X-X
 WARNING: 1-4 contact without 1-(2,3)-4 data for A|501:N02(LIG)       and A|501:N06(LIG)       X-X
 WARNING: 1-4 contact without 1-(2,3)-4 data for A|501:C03(LIG)       and A|501:C07(LIG)       X-X
 WARNING: 1-4 contact without 1-(2,3)-4 data for A|501:C05(LIG)       and A|501:C08(LIG)       X-X
 WARNING: 1-4 contact without 1-(2,3)-4 data for A|501:C05(LIG)       and A|501:C10(LIG)       X-X
 WARNING: 1-4 contact without 1-(2,3)-4 data for A|501:N06(LIG)       and A|501:N09(LIG)       X-X
 WARNING: 1-4 contact without 1-(2,3)-4 data for A|501:C07(LIG)       and A|501:C10(LIG)       X-X
 WARNING: 1-4 contact without 1-(2,3)-4 data for A|501:C08(LIG)       and A|501:C11(LIG)       X-X
 WARNING: 1-4 contact without 1-(2,3)-4 data for B|401:C1(CIT)        and B|401:C4(CIT)        X-X
 WARNING: 1-4 contact without 1-(2,3)-4 data for B|401:C4(CIT)        and B|401:O6(CIT)        X-X

       TERM      weight      number      TNT_fn    Gelly_fn        diff
       BOND       2.000        5653    5575.168    5575.168      -0.000
       ANGL       2.000        7670   11974.029   11974.029       0.000
       IMPR       0.000           0       0.000       0.000       0.000
       TORS       2.000        1968    2940.386    2940.386      -0.000
       TRIG       2.000           0       0.000       0.000       0.000
       PLAN       5.000         963   16176.037   16176.037      -0.000
       CONT       5.000           0    3479.589       0.000    3479.589
       BCOR      10.000        5630  195329.764  196702.438   -1372.675


 ============= Call Gelly geometry functions

   Switching torsion functional form to sinusoidal rather than split harmonic functional.
      if you want this not to happen then rerun with '-torsharm'
      Weight for sinusoidal torsion=     2.000
   BCOR function/gradient will be split between H and non-H atoms (as for any atom pair). Use '-bcorrel_all_in_hydrogens' to change this.

 ----------- Fix B-correlation restraints

 
 Checking for bonds that do not have a bcorrel restraint defined
 We will wipe all existing BCORrels if OCC=0.0 atom(s) is/are involved. This can be' switched off via the '-bcorrel_nowipe_zeroocc' flag.
 We will wipe all existing BCORrels if only non-hydrogen atom(s) with fixed B(iso) are involved. This can be switched off via the '-bcorrel_nowipe_fixb_nonhydrogens' flag.
 We will keep all existing BCORrels if hydrogen atom(s) with fixed B(iso) is/are involved. This can be switched off via the '-bcorrel_wipe_fixb_hydrogens' flag.
 No -bcorrel_missing_sigma found. So will set sigma to     2.5 Ang**2 for any bond with no existing BCORrel
 No -bcorrel_missing_sigma_hydrogens found. So will set sigma to     1.0 Ang**2 for any bond with no existing BCORrel involving hydrogens

 No active BCORels found involving OCC=0.0 atom(s)


 No active BCORels found involving fixed-B (only) non-hydrogen atom(s)

    add a default BCOR for bond A|501:C01(LIG) A|501:N02(LIG)
    add a default BCOR for bond A|501:N02(LIG) A|501:C03(LIG)
    add a default BCOR for bond A|501:C03(LIG) A|501:O04(LIG)
    add a default BCOR for bond A|501:C03(LIG) A|501:C05(LIG)
    add a default BCOR for bond A|501:C05(LIG) A|501:N06(LIG)
    add a default BCOR for bond A|501:N06(LIG) A|501:C07(LIG)
    add a default BCOR for bond A|501:N06(LIG) A|501:C11(LIG)
    add a default BCOR for bond A|501:C07(LIG) A|501:C08(LIG)
    add a default BCOR for bond A|501:C08(LIG) A|501:N09(LIG)
    add a default BCOR for bond A|501:N09(LIG) A|501:C10(LIG)
    add a default BCOR for bond A|501:C10(LIG) A|501:C11(LIG)
    add a default BCOR for bond B|401:C1(CIT) B|401:C2(CIT)
    add a default BCOR for bond B|401:C1(CIT) B|401:O1(CIT)
    add a default BCOR for bond B|401:C1(CIT) B|401:O2(CIT)
    add a default BCOR for bond B|401:C2(CIT) B|401:C3(CIT)
    add a default BCOR for bond B|401:C3(CIT) B|401:C4(CIT)
    add a default BCOR for bond B|401:C3(CIT) B|401:C6(CIT)
    add a default BCOR for bond B|401:C3(CIT) B|401:O7(CIT)
    add a default BCOR for bond B|401:C4(CIT) B|401:C5(CIT)
    add a default BCOR for bond B|401:C5(CIT) B|401:O3(CIT)
    add a default BCOR for bond B|401:C5(CIT) B|401:O4(CIT)
    add a default BCOR for bond B|401:C6(CIT) B|401:O5(CIT)
    add a default BCOR for bond B|401:C6(CIT) B|401:O6(CIT)

 Have added a total of        23 BCORrels for bonds which do not already have a BCORrel


 No bonds (for potential BCORrel addition) were skipped due to both atoms having constant/fixed B(iso)


 ----------- Deactivate hydrogen-free planes if hydrogen atoms present

 A total of       0 planes have been deactivated because they are duplicated hydrogen-free and with-hydrogen forms.

 ----------- Handle peptide omega angle(s)

      Torsion angles split into peptide omega and other
         Number of peptide omega angles is       698
         Number of other torsion is             1270
      Peptide omega angles are monitored rather than directly restrained (PLANE used instead).

 ----------- Fix cis-PRO

 Now apply the fix to get correct restraints for cis PRO residues
   Have got      11 bonds records and      26 angles
   from GEOMETRY cards for the special PROC residue
   Weight for ideal-distance contact term=     4.000

 ----------- Prepare SSBOND/LINK for output PDB file


 ----------- Prepare for QM


 ----------- Prepare symmetry in utility distance restraints


 ############### Setting up Rhofit contacts (if needed)

 No ligand-(fixed protein) contact term will be used

 ############### Working out initial function and gradient


   MaxLik Scaling Cycle 0002
 
 Initial functional value = 5.422485E+05 rms gradient=      781.742
 X-ray contribution to function value = 1.445400E+05
 Geometry contribution (inc ncs)      = 3.977085E+05
 Geometry function summary
     term                           number     weight       rms        GooF      function     fn/numb
     ================================================================================================
     BOND bond lengths (angs)       5653       2.000      0.0141       0.702      5575.17       0.986
     ANGL bond angles (degs)        7670       2.000        1.48       0.884     11974.03       1.561
     TORS split-harmonic tors       1270       0.000       17.10       1.076         0.00       0.000
     SINTOR sinusoidal tors         1270       2.000       17.10                  2290.92       1.804
     PLAN general plane (angs)       963       5.000      0.0367       1.833     16176.04      16.798
     BCORrelations (angs^2)         5653      10.000       5.288       1.887    201315.75      35.612
     CONT (bad contacts)               0       5.000                                 0.00
     IDEAL(ideal-dist contact)      5423       4.000                             28782.84
     CHIRAL (gelly semiharmon)       740       5.000                                14.27
     SIM similarity restraint for NCS and/or TARGET                             131579.44
     OCCSUM restraint on sum of occupanies                                           0.00       as none defined
     DISTAN utility restraints interatomic distance                                  0.00       as none defined
     UTILANGLE utility angle restraints                                              0.00       as none defined
     UTILTOR utility restraints on torsion angles                                    0.00       as none defined
     Peptide omega torsion angles                           7.81 degs - monitor only
 
 WARNING: Have |delta/sigma| deviations > 5.0 sigma. Number of outliers for each term: 
 WARNING:         2 bond angles.  Worst is     8.7 sigs   124.66 degs B|90:NE=CZ=NH1 (ARG)
 WARNING:        19 planes.       Worst is     7.8 sigs     0.16 Angs A|204:C=A|204:CA=A|204:O=A|205:N=A|205:CA=A|205:CD
 WARNING:        12 idealD contacts. Worst     9.9 sigs     1.77 Angs A|234:CE1(HIS)=A|501:C11(LIG)
 WARNING:       150 Bcorrel.      Worst is    16.5 sigs   -41.23 Angs^2 B|401:C6=O5 (CIT)

 Weighted rms      0.0141         1.482  (temporary rms bond and angle for BUSTER to pick up!)
 Weighted intra-residual rms(bond) for StdProtein (excluding H)      0.0145
 Weighted intra-residual rms(bond) for Buffer     (excluding H)      0.0258

 ############### Running initial check


 Initial Geometry Sanity Check

 Check for duplicate bonds: no duplicates found
 
 Check for duplicate or missing bond angles: all are OK
 Check for bond between an atom and itself not involving symmetry:  all are OK
 *** WARNING: There are 8 bad contacts where Delta/sigma is greater than      6.5
 *** WARNING: Looking at each one in turn to see whether it is likely to indicate a setup problem with geometry restraints.

        1 dist=   1.77 idealD=   3.65 sigma=   0.19 abs(Delta/sigma)=    9.91
          Between atoms   A|234:CE1 (HIS)         and   A|501:C11 (LIG)         (C-C)
          occupancies=     1.00        0.72
 [check-049]
 WARNING: Could be a bad contact but may well be an unrecognized link from
          protein to HETATM residue? It is possible restraints have not been
          properly set up.
          If you want BUSTER to regard this as a bond try adding the
          following card before the CRYST1 line in the input pdb file:
LINK         CE1 HIS A 234                 C11 LIG A 501     1555   1555  1.77  

        2 dist=   2.27 idealD=   3.85 sigma=   0.17 abs(Delta/sigma)=    9.59
          Between atoms   A|246:N (GLY)           and   A|501:C03 (LIG)         (N-C)
          occupancies=     1.00        0.72
 [check-049]
          Could be a bad contact but may well be an unrecognized link from
          protein to HETATM residue? It is possible restraints have not been
          properly set up.

        3 dist=   1.90 idealD=   3.66 sigma=   0.20 abs(Delta/sigma)=    8.73
          Between atoms   A|246:CA (GLY)          and   A|501:C03 (LIG)         (C-C)
          occupancies=     1.00        0.72
 [check-049]
 WARNING: Could be a bad contact but may well be an unrecognized link from
          protein to HETATM residue? It is possible restraints have not been
          properly set up.
          If you want BUSTER to regard this as a bond try adding the
          following card before the CRYST1 line in the input pdb file:
LINK         CA  GLY A 246                 C03 LIG A 501     1555   1555  1.90  

        4 dist=   2.15 idealD=   3.73 sigma=   0.19 abs(Delta/sigma)=    8.45
          Between atoms   A|246:CA (GLY)          and   A|501:N02 (LIG)         (C-N)
          occupancies=     1.00        0.72
 [check-049]
          Could be a bad contact but may well be an unrecognized link from
          protein to HETATM residue? It is possible restraints have not been
          properly set up.

        5 dist=   2.05 idealD=   3.65 sigma=   0.19 abs(Delta/sigma)=    8.43
          Between atoms   A|234:CE1 (HIS)         and   A|501:C10 (LIG)         (C-C)
          occupancies=     1.00        0.72
 [check-049]
          Could be a bad contact but may well be an unrecognized link from
          protein to HETATM residue? It is possible restraints have not been
          properly set up.

        6 dist=   2.13 idealD=   3.84 sigma=   0.22 abs(Delta/sigma)=    7.86
          Between atoms   A|246:CA (GLY)          and   A|501:C05 (LIG)         (C-C)
          occupancies=     1.00        0.72
 [check-049]
          Could be a bad contact but may well be an unrecognized link from
          protein to HETATM residue? It is possible restraints have not been
          properly set up.

        7 dist=   2.05 idealD=   3.55 sigma=   0.19 abs(Delta/sigma)=    7.85
          Between atoms   A|244:O (GLN)           and   A|501:C01 (LIG)         (O-C)
          occupancies=     1.00        0.72
 [check-049]
          Could be a bad contact but may well be an unrecognized link from
          protein to HETATM residue? It is possible restraints have not been
          properly set up.

        8 dist=   2.42 idealD=   3.65 sigma=   0.16 abs(Delta/sigma)=    7.58
          Between atoms   A|246:N (GLY)           and   A|501:C01 (LIG)         (N-C)
          occupancies=     1.00        0.72
 [check-049]
          Could be a bad contact but may well be an unrecognized link from
          protein to HETATM residue? It is possible restraints have not been
          properly set up.
 GEOMETRY CHECK OVERALL:  WARN


 ############### Creating geometry/screen report


 ############### Option "tao" - run Tao optimizer

 TAO: Toolkit for Advanced Optimization. Reference: 
     Munson, T., Sarich, J., Wild, S., Benson, S., McInnes, L.C. (2018)
     "TAO User Manual (Revision 3.9)", Mathematics and Computer Science
     Division, Argonne National Laboratory
     ANL/MCS-TM-322, http://www.mcs.anl.gov/petsc/
 
 Have found -keeppdb flag so will write out a .pdb file every 1000000 its during opt
 Will apply TAO optimization 
 Maximum number of iterations      100
 Convergence test on Grms       4.0000 (-glim)
 Limit on rms displacement to original position (rmsD2init): NONE test inactive (-dlim)

    analysing argument no 0 : -autoncs with j=0
    analysing argument no 1 : -hydrogens_input_occ2zero with j=0
    analysing argument no 2 : -hydrogens_output_occ2heavy with j=0
    analysing argument no 3 : -screen with j=0
    analysing argument no 4 : 100 with j=0
    analysing argument no 5 : -screen_sigma with j=0
    analysing argument no 6 : 3.0 with j=0
    analysing argument no 7 : -keeppdb with j=0
    analysing argument no 8 : 1000000 with j=0
    analysing argument no 9 : -taomonitor_verbose with j=0
    found argument starting with "-tao" : -taomonitor_verbose
    analysing argument no 10 : 2 with j=1
    found value after -tao/-options_/-viewer_ argument : "2"
 PETSC/TAO: setting option "-taomonitor_verbose" = "2"


   MaxLik Scaling Cycle 0003
   Iters     Tot_Funct    Xray_Funct    Geom_Funct          Grms     rmsD2init     maxD2init             R         Rfree           LLG       LLGfree     rms(bond)    rms(angle)       Opttag_H
   Iters             K   B_IMPF_FRAG        K_MISS   B_TEMP_MISS   K_IMPF_MISS   B_IMPF_MISS        K_SOLV   B_TEMP_SOLV   K_IMPF_SOLV   B_IMPF_SOLV        B_TEMP     B_TEMP_11     B_TEMP_12     B_TEMP_22     B_TEMP_13     B_TEMP_23     B_TEMP_33      K_WILSON      B_WILSON      FGCYCLES       Scatag_H
   Iters          BOND         ANGLE      IMPROPER       TORSION    SINTORSION      TRIGONAL        PLANAR  BCORRELATION       CONTACT     IDEALDIST        CHIRAL    SIMILARITY       OCCSSUM  UTILDISTANCE     UTILANGLE   UTILTORSION        RHOFIT        ADP-01        ADP-02        ADP-03        ADP-04        ADP-05        ADP-06        ADP-07        ADP-08        ADP-09        ADP-10      RHOFITPC            QM       Funtag_H
       0  5.419856E+05  1.445400E+05  3.974456E+05       777.235  0.000000E+00  0.000000E+00      0.199664      0.209411  0.000000E+00  0.000000E+00     0.0140909       1.48231       Opttag_D
       0       70.3190       1.76328       1.00000  0.000000E+00       1.00000  0.000000E+00      0.842304       46.9116       1.00000       41.5244  0.000000E+00       4.00350  0.000000E+00       4.00350  0.000000E+00  0.000000E+00      -8.00700       71.3295       2.03201        2       Scatag_D
       0       5575.17       11974.0  0.000000E+00  0.000000E+00       2290.92  0.000000E+00       16176.0  2.010529E+05  0.000000E+00       28782.8       14.2741  1.315794E+05 -1.000000E+10 -1.000000E+10 -1.000000E+10 -1.000000E+10 -1.000000E+10  0.000000E+00  0.000000E+00  0.000000E+00  0.000000E+00  0.000000E+00  0.000000E+00  0.000000E+00  0.000000E+00  0.000000E+00  0.000000E+00  0.000000E+00  0.000000E+00       Funtag_D
   Geometry Restraint Deviations SCREEN Information

 

   MaxLik Scaling Cycle 0004

   MaxLik Scaling Cycle 0005

   Wilson plot

   Statistics in resolution bins after refinement cycle 0001
```


|  |  |
| --- | --- |
|  | - Rxpct factors plot- Rxpct factors table- <Log-Lik Gain>- Ln Sigma A- Correlation Coefficients |


```
   Iters     Tot_Funct    Xray_Funct    Geom_Funct          Grms     rmsD2init     maxD2init             R         Rfree           LLG       LLGfree     rms(bond)    rms(angle)
   Iters             K   B_IMPF_FRAG        K_MISS   B_TEMP_MISS   K_IMPF_MISS   B_IMPF_MISS        K_SOLV   B_TEMP_SOLV   K_IMPF_SOLV   B_IMPF_SOLV        B_TEMP     B_TEMP_11     B_TEMP_12     B_TEMP_22     B_TEMP_13     B_TEMP_23     B_TEMP_33      K_WILSON      B_WILSON      FGCYCLES
   Iters          BOND         ANGLE      IMPROPER       TORSION    SINTORSION      TRIGONAL        PLANAR  BCORRELATION       CONTACT     IDEALDIST        CHIRAL    SIMILARITY       OCCSSUM  UTILDISTANCE     UTILANGLE   UTILTORSION        RHOFIT        ADP-01        ADP-02        ADP-03        ADP-04        ADP-05        ADP-06        ADP-07        ADP-08        ADP-09        ADP-10      RHOFITPC            QM
       1  4.436149E+05  1.457558E+05  2.978592E+05       535.884    0.00727569     0.0985893      0.201186      0.210623   -0.00703847   -0.00840685     0.0109330       1.35707       Opttag_D
       1       70.1208       1.81226       1.00000  0.000000E+00       1.00000  0.000000E+00      0.843947       46.3057       1.00000       41.0099  0.000000E+00       4.18747  0.000000E+00       4.18747  0.000000E+00  0.000000E+00      -8.37494       71.3295       2.03201        2       Scatag_D
       1       3356.26       10036.1  0.000000E+00  0.000000E+00       2280.68  0.000000E+00       14096.9  1.194528E+05  0.000000E+00       26891.9       16.2708  1.217283E+05 -1.000000E+10 -1.000000E+10 -1.000000E+10 -1.000000E+10 -1.000000E+10  0.000000E+00  0.000000E+00  0.000000E+00  0.000000E+00  0.000000E+00  0.000000E+00  0.000000E+00  0.000000E+00  0.000000E+00  0.000000E+00  0.000000E+00  0.000000E+00       Funtag_D
   Geometry Restraint Deviations SCREEN Information

 

   MaxLik Scaling Cycle 0006

   Wilson plot

   Statistics in resolution bins after refinement cycle 0002
```


|  |  |
| --- | --- |
|  | - Rxpct factors plot- Rxpct factors table- <Log-Lik Gain>- Ln Sigma A- Correlation Coefficients |


```
   Iters     Tot_Funct    Xray_Funct    Geom_Funct          Grms     rmsD2init     maxD2init             R         Rfree           LLG       LLGfree     rms(bond)    rms(angle)
   Iters             K   B_IMPF_FRAG        K_MISS   B_TEMP_MISS   K_IMPF_MISS   B_IMPF_MISS        K_SOLV   B_TEMP_SOLV   K_IMPF_SOLV   B_IMPF_SOLV        B_TEMP     B_TEMP_11     B_TEMP_12     B_TEMP_22     B_TEMP_13     B_TEMP_23     B_TEMP_33      K_WILSON      B_WILSON      FGCYCLES
   Iters          BOND         ANGLE      IMPROPER       TORSION    SINTORSION      TRIGONAL        PLANAR  BCORRELATION       CONTACT     IDEALDIST        CHIRAL    SIMILARITY       OCCSSUM  UTILDISTANCE     UTILANGLE   UTILTORSION        RHOFIT        ADP-01        ADP-02        ADP-03        ADP-04        ADP-05        ADP-06        ADP-07        ADP-08        ADP-09        ADP-10      RHOFITPC            QM
       2  3.989051E+05  1.461699E+05  2.527352E+05       374.648     0.0127125     0.0516029      0.201904      0.211183   -0.00943391    -0.0105969    0.00947621       1.25735       Opttag_D
       2       70.0311       1.81386       1.00000  0.000000E+00       1.00000  0.000000E+00      0.844310       45.6251       1.00000       41.1601  0.000000E+00       4.25880  0.000000E+00       4.25880  0.000000E+00  0.000000E+00      -8.51759       71.3295       2.03201        1       Scatag_D
       2       2521.43       8615.40  0.000000E+00  0.000000E+00       2271.03  0.000000E+00       12517.8       88241.6  0.000000E+00       25529.3       25.7827  1.130130E+05 -1.000000E+10 -1.000000E+10 -1.000000E+10 -1.000000E+10 -1.000000E+10  0.000000E+00  0.000000E+00  0.000000E+00  0.000000E+00  0.000000E+00  0.000000E+00  0.000000E+00  0.000000E+00  0.000000E+00  0.000000E+00  0.000000E+00  0.000000E+00       Funtag_D
   Geometry Restraint Deviations SCREEN Information

 
       3  3.592128E+05  1.470314E+05  2.121815E+05       335.704     0.0241381     0.0948654      0.203324      0.212561    -0.0144168    -0.0154956     0.0114788       1.20624       Opttag_D
       3       69.9253       1.82845       1.00000  0.000000E+00       1.00000  0.000000E+00      0.844910       44.4548       1.00000       41.4773  0.000000E+00       4.37436  0.000000E+00       4.37436  0.000000E+00  0.000000E+00      -8.74871       71.3295       2.03201        1       Scatag_D
       3       3699.72       7929.13  0.000000E+00  0.000000E+00       2257.28  0.000000E+00       9914.63       68347.5  0.000000E+00       23368.4       80.0706       96584.8 -1.000000E+10 -1.000000E+10 -1.000000E+10 -1.000000E+10 -1.000000E+10  0.000000E+00  0.000000E+00  0.000000E+00  0.000000E+00  0.000000E+00  0.000000E+00  0.000000E+00  0.000000E+00  0.000000E+00  0.000000E+00  0.000000E+00  0.000000E+00       Funtag_D
       4  3.366712E+05  1.477475E+05  1.889237E+05       366.380     0.0340290      0.127359      0.204498      0.213807    -0.0185592    -0.0203455     0.0134251       1.39411       Opttag_D
       4       69.8439       1.84193       1.00000  0.000000E+00       1.00000  0.000000E+00      0.845167       43.5931       1.00000       41.7223  0.000000E+00       4.45267  0.000000E+00       4.45267  0.000000E+00  0.000000E+00      -8.90533       71.3295       2.03201        1       Scatag_D
       4       5060.74       10591.4  0.000000E+00  0.000000E+00       2249.78  0.000000E+00       8227.47       57372.3  0.000000E+00       21991.3       144.171       83286.5 -1.000000E+10 -1.000000E+10 -1.000000E+10 -1.000000E+10 -1.000000E+10  0.000000E+00  0.000000E+00  0.000000E+00  0.000000E+00  0.000000E+00  0.000000E+00  0.000000E+00  0.000000E+00  0.000000E+00  0.000000E+00  0.000000E+00  0.000000E+00       Funtag_D
       5  3.175399E+05  1.480995E+05  1.694404E+05       259.732     0.0406584      0.145372      0.205161      0.214602    -0.0205952    -0.0229484     0.0128986       1.25688       Opttag_D
       5       69.7827       1.84020       1.00000  0.000000E+00       1.00000  0.000000E+00      0.845076       43.0184       1.00000       41.9535  0.000000E+00       4.49177  0.000000E+00       4.49177  0.000000E+00  0.000000E+00      -8.98355       71.3295       2.03201        1       Scatag_D
       5       4671.58       8608.99  0.000000E+00  0.000000E+00       2246.20  0.000000E+00       7391.41       50029.5  0.000000E+00       21256.9       157.694       75078.2 -1.000000E+10 -1.000000E+10 -1.000000E+10 -1.000000E+10 -1.000000E+10  0.000000E+00  0.000000E+00  0.000000E+00  0.000000E+00  0.000000E+00  0.000000E+00  0.000000E+00  0.000000E+00  0.000000E+00  0.000000E+00  0.000000E+00  0.000000E+00       Funtag_D
       6  2.952684E+05  1.488677E+05  1.464007E+05       272.565     0.0546317      0.210122      0.206575      0.216247    -0.0250387    -0.0283233     0.0120208       1.33236       Opttag_D
       6       69.6715       1.84265       1.00000  0.000000E+00       1.00000  0.000000E+00      0.844877       41.9391       1.00000       42.4053  0.000000E+00       4.56126  0.000000E+00       4.56126  0.000000E+00  0.000000E+00      -9.12251       71.3295       2.03201        1       Scatag_D
       6       4057.39       9673.93  0.000000E+00  0.000000E+00       2243.41  0.000000E+00       6187.90       41987.6  0.000000E+00       20104.6       151.718       61994.1 -1.000000E+10 -1.000000E+10 -1.000000E+10 -1.000000E+10 -1.000000E+10  0.000000E+00  0.000000E+00  0.000000E+00  0.000000E+00  0.000000E+00  0.000000E+00  0.000000E+00  0.000000E+00  0.000000E+00  0.000000E+00  0.000000E+00  0.000000E+00       Funtag_D
       7  2.837711E+05  1.494009E+05  1.343702E+05       292.280     0.0633304      0.257848      0.207529      0.217290    -0.0281226    -0.0322364     0.0115939       1.39290       Opttag_D
       7       69.6025       1.85021       1.00000  0.000000E+00       1.00000  0.000000E+00      0.844805       41.3548       1.00000       42.6317  0.000000E+00       4.60042  0.000000E+00       4.60042  0.000000E+00  0.000000E+00      -9.20084       71.3295       2.03201        1       Scatag_D
       7       3774.29       10573.1  0.000000E+00  0.000000E+00       2243.95  0.000000E+00       5702.48       36598.7  0.000000E+00       19499.9       128.181       55849.7 -1.000000E+10 -1.000000E+10 -1.000000E+10 -1.000000E+10 -1.000000E+10  0.000000E+00  0.000000E+00  0.000000E+00  0.000000E+00  0.000000E+00  0.000000E+00  0.000000E+00  0.000000E+00  0.000000E+00  0.000000E+00  0.000000E+00  0.000000E+00       Funtag_D
       8  2.744933E+05  1.495252E+05  1.249681E+05       191.042     0.0665085      0.284917      0.207805      0.217603    -0.0288419    -0.0332948     0.0106842       1.27895       Opttag_D
       8       69.5658       1.84674       1.00000  0.000000E+00       1.00000  0.000000E+00      0.844691       41.1321       1.00000       42.7204  0.000000E+00       4.60974  0.000000E+00       4.60974  0.000000E+00  0.000000E+00      -9.21949       71.3295       2.03201        1       Scatag_D
       8       3205.27       8913.92  0.000000E+00  0.000000E+00       2242.74  0.000000E+00       5572.42       32707.8  0.000000E+00       19241.2       100.841       52983.9 -1.000000E+10 -1.000000E+10 -1.000000E+10 -1.000000E+10 -1.000000E+10  0.000000E+00  0.000000E+00  0.000000E+00  0.000000E+00  0.000000E+00  0.000000E+00  0.000000E+00  0.000000E+00  0.000000E+00  0.000000E+00  0.000000E+00  0.000000E+00       Funtag_D
       9  2.648233E+05  1.497651E+05  1.150582E+05       157.725     0.0721082      0.340968      0.208285      0.218139    -0.0302294    -0.0353535    0.00988241       1.21762       Opttag_D
       9       69.5105       1.84441       1.00000  0.000000E+00       1.00000  0.000000E+00      0.844522       40.7736       1.00000       42.8449  0.000000E+00       4.62486  0.000000E+00       4.62486  0.000000E+00  0.000000E+00      -9.24973       71.3295       2.03201        1       Scatag_D
       9       2742.23       8079.47  0.000000E+00  0.000000E+00       2241.42  0.000000E+00       5356.66       29464.2  0.000000E+00       18794.1       73.0262       48307.1 -1.000000E+10 -1.000000E+10 -1.000000E+10 -1.000000E+10 -1.000000E+10  0.000000E+00  0.000000E+00  0.000000E+00  0.000000E+00  0.000000E+00  0.000000E+00  0.000000E+00  0.000000E+00  0.000000E+00  0.000000E+00  0.000000E+00  0.000000E+00       Funtag_D
      10  2.580866E+05  1.500492E+05  1.080374E+05       179.742     0.0777740      0.396134      0.208823      0.218830    -0.0318729    -0.0377339    0.00963145       1.23347       Opttag_D
      10       69.4688       1.84623       1.00000  0.000000E+00       1.00000  0.000000E+00      0.844348       40.4190       1.00000       42.9735  0.000000E+00       4.64045  0.000000E+00       4.64045  0.000000E+00  0.000000E+00      -9.28089       71.3295       2.03201        1       Scatag_D
      10       2604.72       8291.19  0.000000E+00  0.000000E+00       2242.20  0.000000E+00       5112.44       26694.6  0.000000E+00       18372.5       69.1561       44650.6 -1.000000E+10 -1.000000E+10 -1.000000E+10 -1.000000E+10 -1.000000E+10  0.000000E+00  0.000000E+00  0.000000E+00  0.000000E+00  0.000000E+00  0.000000E+00  0.000000E+00  0.000000E+00  0.000000E+00  0.000000E+00  0.000000E+00  0.000000E+00       Funtag_D
      11  2.498255E+05  1.505686E+05       99256.9       179.197     0.0874728      0.487671      0.209788      0.220060    -0.0348772    -0.0417700    0.00948108       1.21548       Opttag_D
      11       69.4133       1.85249       1.00000  0.000000E+00       1.00000  0.000000E+00      0.844022       39.8121       1.00000       43.2136  0.000000E+00       4.66603  0.000000E+00       4.66603  0.000000E+00  0.000000E+00      -9.33206       71.3295       2.03201        1       Scatag_D
      11       2524.02       8051.15  0.000000E+00  0.000000E+00       2243.92  0.000000E+00       4743.14       23573.4  0.000000E+00       17749.4       73.1244       40298.7 -1.000000E+10 -1.000000E+10 -1.000000E+10 -1.000000E+10 -1.000000E+10  0.000000E+00  0.000000E+00  0.000000E+00  0.000000E+00  0.000000E+00  0.000000E+00  0.000000E+00  0.000000E+00  0.000000E+00  0.000000E+00  0.000000E+00  0.000000E+00       Funtag_D
      12  2.446424E+05  1.509214E+05       93721.1       216.481     0.0936414      0.541071      0.210462      0.220896    -0.0369176    -0.0446565    0.00953220       1.25627       Opttag_D
      12       69.3797       1.85585       1.00000  0.000000E+00       1.00000  0.000000E+00      0.843719       39.3851       1.00000       43.4009  0.000000E+00       4.68949  0.000000E+00       4.68949  0.000000E+00  0.000000E+00      -9.37898       71.3295       2.03201        1       Scatag_D
      12       2551.31       8600.57  0.000000E+00  0.000000E+00       2247.57  0.000000E+00       4454.09       20637.5  0.000000E+00       17317.5       78.0178       37834.5 -1.000000E+10 -1.000000E+10 -1.000000E+10 -1.000000E+10 -1.000000E+10  0.000000E+00  0.000000E+00  0.000000E+00  0.000000E+00  0.000000E+00  0.000000E+00  0.000000E+00  0.000000E+00  0.000000E+00  0.000000E+00  0.000000E+00  0.000000E+00       Funtag_D
      13  2.399390E+05  1.509122E+05       89026.8       122.588     0.0938149      0.540106      0.210504      0.220949    -0.0368648    -0.0448241    0.00901038       1.15461       Opttag_D
      13       69.3705       1.85026       1.00000  0.000000E+00       1.00000  0.000000E+00      0.843561       39.2984       1.00000       43.4566  0.000000E+00       4.69605  0.000000E+00       4.69605  0.000000E+00  0.000000E+00      -9.39210       71.3295       2.03201        1       Scatag_D
      13       2279.63       7264.92  0.000000E+00  0.000000E+00       2246.27  0.000000E+00       4329.46       19085.6  0.000000E+00       17186.0       78.8311       36556.0 -1.000000E+10 -1.000000E+10 -1.000000E+10 -1.000000E+10 -1.000000E+10  0.000000E+00  0.000000E+00  0.000000E+00  0.000000E+00  0.000000E+00  0.000000E+00  0.000000E+00  0.000000E+00  0.000000E+00  0.000000E+00  0.000000E+00  0.000000E+00       Funtag_D
      14  2.357606E+05  1.509565E+05       84804.1       101.349     0.0950526      0.541848      0.210667      0.221137    -0.0371209    -0.0455354    0.00874573       1.12697       Opttag_D
      14       69.3523       1.84282       1.00000  0.000000E+00       1.00000  0.000000E+00      0.843250       39.1103       1.00000       43.5704  0.000000E+00       4.70824  0.000000E+00       4.70824  0.000000E+00  0.000000E+00      -9.41648       71.3295       2.03201        1       Scatag_D
      14       2147.68       6921.27  0.000000E+00  0.000000E+00       2244.20  0.000000E+00       4129.35       17828.7  0.000000E+00       16939.6       77.3475       34515.9 -1.000000E+10 -1.000000E+10 -1.000000E+10 -1.000000E+10 -1.000000E+10  0.000000E+00  0.000000E+00  0.000000E+00  0.000000E+00  0.000000E+00  0.000000E+00  0.000000E+00  0.000000E+00  0.000000E+00  0.000000E+00  0.000000E+00  0.000000E+00       Funtag_D
      15  2.324503E+05  1.511260E+05       81324.3       143.332     0.0982423      0.550861      0.211034      0.221630    -0.0381014    -0.0473303    0.00867547       1.14782       Opttag_D
      15       69.3297       1.83937       1.00000  0.000000E+00       1.00000  0.000000E+00      0.842810       38.8309       1.00000       43.7310  0.000000E+00       4.72371  0.000000E+00       4.72371  0.000000E+00  0.000000E+00      -9.44742       71.3295       2.03201        1       Scatag_D
      15       2113.31       7179.70  0.000000E+00  0.000000E+00       2244.31  0.000000E+00       3925.13       16693.7  0.000000E+00       16607.8       71.9691       32488.3 -1.000000E+10 -1.000000E+10 -1.000000E+10 -1.000000E+10 -1.000000E+10  0.000000E+00  0.000000E+00  0.000000E+00  0.000000E+00  0.000000E+00  0.000000E+00  0.000000E+00  0.000000E+00  0.000000E+00  0.000000E+00  0.000000E+00  0.000000E+00       Funtag_D
      16  2.285734E+05  1.514131E+05       77160.3       109.288      0.103091      0.561135      0.211581      0.222390    -0.0397616    -0.0500875    0.00841648       1.10753       Opttag_D
      16       69.3080       1.84086       1.00000  0.000000E+00       1.00000  0.000000E+00      0.842279       38.4867       1.00000       43.9203  0.000000E+00       4.74205  0.000000E+00       4.74205  0.000000E+00  0.000000E+00      -9.48410       71.3295       2.03201        1       Scatag_D
      16       1989.02       6684.51  0.000000E+00  0.000000E+00       2244.07  0.000000E+00       3741.54       15426.3  0.000000E+00       16205.6       65.1070       30804.2 -1.000000E+10 -1.000000E+10 -1.000000E+10 -1.000000E+10 -1.000000E+10  0.000000E+00  0.000000E+00  0.000000E+00  0.000000E+00  0.000000E+00  0.000000E+00  0.000000E+00  0.000000E+00  0.000000E+00  0.000000E+00  0.000000E+00  0.000000E+00       Funtag_D
      17  2.255775E+05  1.516271E+05       73950.4       115.183      0.106821      0.565859      0.211973      0.222915    -0.0409997    -0.0520800    0.00813464       1.11075       Opttag_D
      17       69.3012       1.84353       1.00000  0.000000E+00       1.00000  0.000000E+00      0.841846       38.2485       1.00000       44.0527  0.000000E+00       4.75301  0.000000E+00       4.75301  0.000000E+00  0.000000E+00      -9.50602       71.3295       2.03201        1       Scatag_D
      17       1858.03       6723.42  0.000000E+00  0.000000E+00       2242.63  0.000000E+00       3640.39       13852.8  0.000000E+00       15871.1       61.8848       29700.1 -1.000000E+10 -1.000000E+10 -1.000000E+10 -1.000000E+10 -1.000000E+10  0.000000E+00  0.000000E+00  0.000000E+00  0.000000E+00  0.000000E+00  0.000000E+00  0.000000E+00  0.000000E+00  0.000000E+00  0.000000E+00  0.000000E+00  0.000000E+00       Funtag_D
      18  2.230342E+05  1.517961E+05       71238.0       90.9967      0.109812      0.610012      0.212274      0.223284    -0.0419774    -0.0536646    0.00791560       1.08589       Opttag_D
      18       69.3070       1.84694       1.00000  0.000000E+00       1.00000  0.000000E+00      0.841479       38.0683       1.00000       44.1567  0.000000E+00       4.76112  0.000000E+00       4.76112  0.000000E+00  0.000000E+00      -9.52224       71.3295       2.03201        1       Scatag_D
      18       1759.32       6425.89  0.000000E+00  0.000000E+00       2240.98  0.000000E+00       3569.59       12886.9  0.000000E+00       15568.9       62.7998       28723.7 -1.000000E+10 -1.000000E+10 -1.000000E+10 -1.000000E+10 -1.000000E+10  0.000000E+00  0.000000E+00  0.000000E+00  0.000000E+00  0.000000E+00  0.000000E+00  0.000000E+00  0.000000E+00  0.000000E+00  0.000000E+00  0.000000E+00  0.000000E+00       Funtag_D
      19  2.211825E+05  1.518616E+05       69320.8       74.7719      0.111015      0.628983      0.212386      0.223401    -0.0423564    -0.0543223    0.00770939       1.06723       Opttag_D
      19       69.3210       1.84883       1.00000  0.000000E+00       1.00000  0.000000E+00      0.841290       37.9902       1.00000       44.2024  0.000000E+00       4.76581  0.000000E+00       4.76581  0.000000E+00  0.000000E+00      -9.53161       71.3295       2.03201        1       Scatag_D
      19       1668.85       6206.94  0.000000E+00  0.000000E+00       2237.97  0.000000E+00       3525.93       12412.1  0.000000E+00       15366.2       66.4757       27836.4 -1.000000E+10 -1.000000E+10 -1.000000E+10 -1.000000E+10 -1.000000E+10  0.000000E+00  0.000000E+00  0.000000E+00  0.000000E+00  0.000000E+00  0.000000E+00  0.000000E+00  0.000000E+00  0.000000E+00  0.000000E+00  0.000000E+00  0.000000E+00       Funtag_D
      20  2.188820E+05  1.519984E+05       66883.7       89.1244      0.113392      0.659923      0.212623      0.223653    -0.0431473    -0.0555124    0.00761659       1.06098       Opttag_D
      20       69.3567       1.85367       1.00000  0.000000E+00       1.00000  0.000000E+00      0.840957       37.8322       1.00000       44.2985  0.000000E+00       4.77606  0.000000E+00       4.77606  0.000000E+00  0.000000E+00      -9.55211       71.3295       2.03201        1       Scatag_D
      20       1628.92       6134.43  0.000000E+00  0.000000E+00       2232.00  0.000000E+00       3444.41       11873.1  0.000000E+00       14992.3       73.5282       26504.9 -1.000000E+10 -1.000000E+10 -1.000000E+10 -1.000000E+10 -1.000000E+10  0.000000E+00  0.000000E+00  0.000000E+00  0.000000E+00  0.000000E+00  0.000000E+00  0.000000E+00  0.000000E+00  0.000000E+00  0.000000E+00  0.000000E+00  0.000000E+00       Funtag_D
      21  2.172191E+05  1.522176E+05       65001.5       119.799      0.116822      0.693611      0.213001      0.224089    -0.0444153    -0.0574779    0.00768588       1.07771       Opttag_D
      21       69.3955       1.86205       1.00000  0.000000E+00       1.00000  0.000000E+00      0.840572       37.6272       1.00000       44.4305  0.000000E+00       4.79058  0.000000E+00       4.79058  0.000000E+00  0.000000E+00      -9.58115       71.3295       2.03201        1       Scatag_D
      21       1658.69       6329.39  0.000000E+00  0.000000E+00       2228.05  0.000000E+00       3349.58       11058.5  0.000000E+00       14626.8       72.0249       25678.5 -1.000000E+10 -1.000000E+10 -1.000000E+10 -1.000000E+10 -1.000000E+10  0.000000E+00  0.000000E+00  0.000000E+00  0.000000E+00  0.000000E+00  0.000000E+00  0.000000E+00  0.000000E+00  0.000000E+00  0.000000E+00  0.000000E+00  0.000000E+00       Funtag_D
      22  2.156321E+05  1.522851E+05       63347.0       67.6044      0.117913      0.699807      0.213125      0.224269    -0.0448057    -0.0581154    0.00750552       1.04164       Opttag_D
      22       69.4067       1.86410       1.00000  0.000000E+00       1.00000  0.000000E+00      0.840435       37.5562       1.00000       44.4775  0.000000E+00       4.79479  0.000000E+00       4.79479  0.000000E+00  0.000000E+00      -9.58957       71.3295       2.03201        1       Scatag_D
      22       1581.75       5912.86  0.000000E+00  0.000000E+00       2226.54  0.000000E+00       3297.62       10471.3  0.000000E+00       14505.3       65.8464       25285.8 -1.000000E+10 -1.000000E+10 -1.000000E+10 -1.000000E+10 -1.000000E+10  0.000000E+00  0.000000E+00  0.000000E+00  0.000000E+00  0.000000E+00  0.000000E+00  0.000000E+00  0.000000E+00  0.000000E+00  0.000000E+00  0.000000E+00  0.000000E+00       Funtag_D
      23  2.140520E+05  1.524559E+05       61596.0       60.1453      0.120696      0.716336      0.213429      0.224710    -0.0457939    -0.0596656    0.00744381       1.03291       Opttag_D
      23       69.4313       1.87037       1.00000  0.000000E+00       1.00000  0.000000E+00      0.840111       37.4034       1.00000       44.5814  0.000000E+00       4.80187  0.000000E+00       4.80187  0.000000E+00  0.000000E+00      -9.60373       71.3295       2.03201        1       Scatag_D
      23       1555.85       5814.13  0.000000E+00  0.000000E+00       2224.71  0.000000E+00       3199.88       9888.13  0.000000E+00       14257.1       57.2026       24599.0 -1.000000E+10 -1.000000E+10 -1.000000E+10 -1.000000E+10 -1.000000E+10  0.000000E+00  0.000000E+00  0.000000E+00  0.000000E+00  0.000000E+00  0.000000E+00  0.000000E+00  0.000000E+00  0.000000E+00  0.000000E+00  0.000000E+00  0.000000E+00       Funtag_D
      24  2.127695E+05  1.526779E+05       60091.6       87.7789      0.124392      0.747435      0.213803      0.225277    -0.0470780    -0.0616920    0.00744059       1.04249       Opttag_D
      24       69.4712       1.88105       1.00000  0.000000E+00       1.00000  0.000000E+00      0.839699       37.2412       1.00000       44.6955  0.000000E+00       4.80881  0.000000E+00       4.80881  0.000000E+00  0.000000E+00      -9.61762       71.3295       2.03201        1       Scatag_D
      24       1554.50       5922.49  0.000000E+00  0.000000E+00       2223.48  0.000000E+00       3110.25       9412.22  0.000000E+00       13952.4       54.3908       23861.9 -1.000000E+10 -1.000000E+10 -1.000000E+10 -1.000000E+10 -1.000000E+10  0.000000E+00  0.000000E+00  0.000000E+00  0.000000E+00  0.000000E+00  0.000000E+00  0.000000E+00  0.000000E+00  0.000000E+00  0.000000E+00  0.000000E+00  0.000000E+00       Funtag_D
      25  2.110916E+05  1.530375E+05       58054.1       70.9659      0.130249      0.802253      0.214387      0.226178    -0.0491578    -0.0648584    0.00739628       1.02536       Opttag_D
      25       69.5420       1.90159       1.00000  0.000000E+00       1.00000  0.000000E+00      0.839114       37.0287       1.00000       44.8523  0.000000E+00       4.81694  0.000000E+00       4.81694  0.000000E+00  0.000000E+00      -9.63388       71.3295       2.03201        1       Scatag_D
      25       1536.05       5729.51  0.000000E+00  0.000000E+00       2222.67  0.000000E+00       3005.56       9021.23  0.000000E+00       13534.9       53.8266       22950.4 -1.000000E+10 -1.000000E+10 -1.000000E+10 -1.000000E+10 -1.000000E+10  0.000000E+00  0.000000E+00  0.000000E+00  0.000000E+00  0.000000E+00  0.000000E+00  0.000000E+00  0.000000E+00  0.000000E+00  0.000000E+00  0.000000E+00  0.000000E+00       Funtag_D
      26  2.096592E+05  1.532465E+05       56412.7       80.4737      0.134149      0.836802      0.214707      0.226757    -0.0503666    -0.0669027    0.00728668       1.02646       Opttag_D
      26       69.6046       1.91666       1.00000  0.000000E+00       1.00000  0.000000E+00      0.838743       36.9290       1.00000       44.9409  0.000000E+00       4.81922  0.000000E+00       4.81922  0.000000E+00  0.000000E+00      -9.63843       71.3295       2.03201        1       Scatag_D
      26       1490.86       5741.75  0.000000E+00  0.000000E+00       2222.90  0.000000E+00       2953.90       8476.16  0.000000E+00       13253.2       57.0851       22216.8 -1.000000E+10 -1.000000E+10 -1.000000E+10 -1.000000E+10 -1.000000E+10  0.000000E+00  0.000000E+00  0.000000E+00  0.000000E+00  0.000000E+00  0.000000E+00  0.000000E+00  0.000000E+00  0.000000E+00  0.000000E+00  0.000000E+00  0.000000E+00       Funtag_D
      27  2.082822E+05  1.533574E+05       54924.8       60.7899      0.136801      0.859090      0.214863      0.227173    -0.0510079    -0.0682742    0.00710532       1.01128       Opttag_D
      27       69.6630       1.92756       1.00000  0.000000E+00       1.00000  0.000000E+00      0.838489       36.8923       1.00000       44.9944  0.000000E+00       4.81910  0.000000E+00       4.81910  0.000000E+00  0.000000E+00      -9.63820       71.3295       2.03201        1       Scatag_D
      27       1417.57       5573.22  0.000000E+00  0.000000E+00       2222.26  0.000000E+00       2924.53       8103.43  0.000000E+00       13040.7       61.6998       21581.4 -1.000000E+10 -1.000000E+10 -1.000000E+10 -1.000000E+10 -1.000000E+10  0.000000E+00  0.000000E+00  0.000000E+00  0.000000E+00  0.000000E+00  0.000000E+00  0.000000E+00  0.000000E+00  0.000000E+00  0.000000E+00  0.000000E+00  0.000000E+00       Funtag_D
      28  2.073369E+05  1.533535E+05       53983.3       53.8083      0.137751      0.866267      0.214843      0.227333    -0.0509858    -0.0686931    0.00697238       1.00030       Opttag_D
      28       69.7059       1.93156       1.00000  0.000000E+00       1.00000  0.000000E+00      0.838380       36.9078       1.00000       45.0119  0.000000E+00       4.81607  0.000000E+00       4.81607  0.000000E+00  0.000000E+00      -9.63214       71.3295       2.03201        1       Scatag_D
      28       1365.02       5452.88  0.000000E+00  0.000000E+00       2220.59  0.000000E+00       2906.21       8002.86  0.000000E+00       12913.5       63.7537       21058.6 -1.000000E+10 -1.000000E+10 -1.000000E+10 -1.000000E+10 -1.000000E+10  0.000000E+00  0.000000E+00  0.000000E+00  0.000000E+00  0.000000E+00  0.000000E+00  0.000000E+00  0.000000E+00  0.000000E+00  0.000000E+00  0.000000E+00  0.000000E+00       Funtag_D
      29  2.064466E+05  1.533412E+05       53105.4       56.2431      0.138760      0.873067      0.214812      0.227514    -0.0509145    -0.0691176    0.00688430      0.993140       Opttag_D
      29       69.7612       1.93605       1.00000  0.000000E+00       1.00000  0.000000E+00      0.838259       36.9246       1.00000       45.0394  0.000000E+00       4.81245  0.000000E+00       4.81245  0.000000E+00  0.000000E+00      -9.62489       71.3295       2.03201        1       Scatag_D
      29       1330.75       5375.05  0.000000E+00  0.000000E+00       2218.05  0.000000E+00       2876.42       7904.28  0.000000E+00       12772.9       61.0391       20567.0 -1.000000E+10 -1.000000E+10 -1.000000E+10 -1.000000E+10 -1.000000E+10  0.000000E+00  0.000000E+00  0.000000E+00  0.000000E+00  0.000000E+00  0.000000E+00  0.000000E+00  0.000000E+00  0.000000E+00  0.000000E+00  0.000000E+00  0.000000E+00       Funtag_D
      30  2.054362E+05  1.534616E+05       51974.5       50.6093      0.141520      0.892917      0.214978      0.227986    -0.0516111    -0.0706139    0.00683747      0.985549       Opttag_D
      30       69.8461       1.95012       1.00000  0.000000E+00       1.00000  0.000000E+00      0.838005       36.8919       1.00000       45.1115  0.000000E+00       4.81146  0.000000E+00       4.81146  0.000000E+00  0.000000E+00      -9.62291       71.3295       2.03201        1       Scatag_D
      30       1312.71       5293.20  0.000000E+00  0.000000E+00       2215.75  0.000000E+00       2816.60       7553.42  0.000000E+00       12554.8       53.9273       20174.1 -1.000000E+10 -1.000000E+10 -1.000000E+10 -1.000000E+10 -1.000000E+10  0.000000E+00  0.000000E+00  0.000000E+00  0.000000E+00  0.000000E+00  0.000000E+00  0.000000E+00  0.000000E+00  0.000000E+00  0.000000E+00  0.000000E+00  0.000000E+00       Funtag_D
      31  2.044956E+05  1.536864E+05       50809.2       54.8041      0.145458      0.920860      0.215293      0.228614    -0.0529112    -0.0727715    0.00684928      0.982978       Opttag_D
      31       69.9533       1.97269       1.00000  0.000000E+00       1.00000  0.000000E+00      0.837707       36.8330       1.00000       45.2043  0.000000E+00       4.81238  0.000000E+00       4.81238  0.000000E+00  0.000000E+00      -9.62476       71.3295       2.03201        1       Scatag_D
      31       1317.25       5265.61  0.000000E+00  0.000000E+00       2213.82  0.000000E+00       2728.37       7147.33  0.000000E+00       12292.4       47.2935       19797.1 -1.000000E+10 -1.000000E+10 -1.000000E+10 -1.000000E+10 -1.000000E+10  0.000000E+00  0.000000E+00  0.000000E+00  0.000000E+00  0.000000E+00  0.000000E+00  0.000000E+00  0.000000E+00  0.000000E+00  0.000000E+00  0.000000E+00  0.000000E+00       Funtag_D
      32  2.037998E+05  1.539035E+05       49896.4       59.9433      0.149193      0.946874      0.215583      0.229146    -0.0541667    -0.0747758    0.00681852      0.982879       Opttag_D
      32       70.0542       1.99520       1.00000  0.000000E+00       1.00000  0.000000E+00      0.837447       36.7883       1.00000       45.2811  0.000000E+00       4.81333  0.000000E+00       4.81333  0.000000E+00  0.000000E+00      -9.62666       71.3295       2.03201        1       Scatag_D
      32       1305.44       5264.55  0.000000E+00  0.000000E+00       2212.95  0.000000E+00       2645.97       6897.95  0.000000E+00       12061.1       46.2106       19462.2 -1.000000E+10 -1.000000E+10 -1.000000E+10 -1.000000E+10 -1.000000E+10  0.000000E+00  0.000000E+00  0.000000E+00  0.000000E+00  0.000000E+00  0.000000E+00  0.000000E+00  0.000000E+00  0.000000E+00  0.000000E+00  0.000000E+00  0.000000E+00       Funtag_D
      33  2.031579E+05  1.540308E+05       49127.1       41.4746      0.151345      0.962079      0.215745      0.229420    -0.0549032    -0.0759147    0.00672307      0.973509       Opttag_D
      33       70.1151       2.00912       1.00000  0.000000E+00       1.00000  0.000000E+00      0.837310       36.7709       1.00000       45.3191  0.000000E+00       4.81427  0.000000E+00       4.81427  0.000000E+00  0.000000E+00      -9.62854       71.3295       2.03201        1       Scatag_D
      33       1269.15       5164.65  0.000000E+00  0.000000E+00       2211.94  0.000000E+00       2591.24       6757.02  0.000000E+00       11923.6       48.1230       19161.5 -1.000000E+10 -1.000000E+10 -1.000000E+10 -1.000000E+10 -1.000000E+10  0.000000E+00  0.000000E+00  0.000000E+00  0.000000E+00  0.000000E+00  0.000000E+00  0.000000E+00  0.000000E+00  0.000000E+00  0.000000E+00  0.000000E+00  0.000000E+00       Funtag_D
      34  2.024123E+05  1.541293E+05       48283.1       37.0907      0.153515      0.977334      0.215839      0.229626    -0.0554727    -0.0769215    0.00664996      0.967971       Opttag_D
      34       70.1910       2.02366       1.00000  0.000000E+00       1.00000  0.000000E+00      0.837185       36.7800       1.00000       45.3487  0.000000E+00       4.81331  0.000000E+00       4.81331  0.000000E+00  0.000000E+00      -9.62662       71.3295       2.03201        1       Scatag_D
      34       1241.70       5106.05  0.000000E+00  0.000000E+00       2210.46  0.000000E+00       2538.23       6611.74  0.000000E+00       11767.1       53.6169       18754.2 -1.000000E+10 -1.000000E+10 -1.000000E+10 -1.000000E+10 -1.000000E+10  0.000000E+00  0.000000E+00  0.000000E+00  0.000000E+00  0.000000E+00  0.000000E+00  0.000000E+00  0.000000E+00  0.000000E+00  0.000000E+00  0.000000E+00  0.000000E+00       Funtag_D
      35  2.017987E+05  1.542547E+05       47544.1       78.1814      0.157049       1.00097      0.215925      0.229947    -0.0561981    -0.0784402    0.00668312      0.981970       Opttag_D
      35       70.3291       2.04736       1.00000  0.000000E+00       1.00000  0.000000E+00      0.836994       36.8205       1.00000       45.4023  0.000000E+00       4.80912  0.000000E+00       4.80912  0.000000E+00  0.000000E+00      -9.61824       71.3295       2.03201        1       Scatag_D
      35       1254.11       5254.82  0.000000E+00  0.000000E+00       2207.37  0.000000E+00       2488.60       6420.22  0.000000E+00       11536.0       55.2855       18327.7 -1.000000E+10 -1.000000E+10 -1.000000E+10 -1.000000E+10 -1.000000E+10  0.000000E+00  0.000000E+00  0.000000E+00  0.000000E+00  0.000000E+00  0.000000E+00  0.000000E+00  0.000000E+00  0.000000E+00  0.000000E+00  0.000000E+00  0.000000E+00       Funtag_D
      36  2.011024E+05  1.543256E+05       46776.8       39.1715      0.159155       1.01450      0.215971      0.230142    -0.0566085    -0.0793483    0.00651984      0.958859       Opttag_D
      36       70.4175       2.06228       1.00000  0.000000E+00       1.00000  0.000000E+00      0.836887       36.8537       1.00000       45.4359  0.000000E+00       4.80579  0.000000E+00       4.80579  0.000000E+00  0.000000E+00      -9.61158       71.3295       2.03201        1       Scatag_D
      36       1193.58       5010.38  0.000000E+00  0.000000E+00       2206.35  0.000000E+00       2470.85       6271.38  0.000000E+00       11411.3       52.4611       18160.4 -1.000000E+10 -1.000000E+10 -1.000000E+10 -1.000000E+10 -1.000000E+10  0.000000E+00  0.000000E+00  0.000000E+00  0.000000E+00  0.000000E+00  0.000000E+00  0.000000E+00  0.000000E+00  0.000000E+00  0.000000E+00  0.000000E+00  0.000000E+00       Funtag_D
      37  2.006645E+05  1.543598E+05       46304.8       33.0584      0.160534       1.02331      0.215977      0.230254    -0.0568062    -0.0798905    0.00643591      0.953767       Opttag_D
      37       70.4844       2.07257       1.00000  0.000000E+00       1.00000  0.000000E+00      0.836814       36.8871       1.00000       45.4595  0.000000E+00       4.80225  0.000000E+00       4.80225  0.000000E+00  0.000000E+00      -9.60450       71.3295       2.03201        1       Scatag_D
      37       1163.05       4957.31  0.000000E+00  0.000000E+00       2205.33  0.000000E+00       2463.21       6130.44  0.000000E+00       11330.1       47.3996       18007.9 -1.000000E+10 -1.000000E+10 -1.000000E+10 -1.000000E+10 -1.000000E+10  0.000000E+00  0.000000E+00  0.000000E+00  0.000000E+00  0.000000E+00  0.000000E+00  0.000000E+00  0.000000E+00  0.000000E+00  0.000000E+00  0.000000E+00  0.000000E+00       Funtag_D
      38  2.001575E+05  1.544458E+05       45711.8       41.8821      0.163132       1.03992      0.216017      0.230487    -0.0573034    -0.0809730    0.00639804      0.952013       Opttag_D
      38       70.6051       2.09313       1.00000  0.000000E+00       1.00000  0.000000E+00      0.836691       36.9454       1.00000       45.4979  0.000000E+00       4.79669  0.000000E+00       4.79669  0.000000E+00  0.000000E+00      -9.59337       71.3295       2.03201        1       Scatag_D
      38       1149.40       4939.09  0.000000E+00  0.000000E+00       2203.77  0.000000E+00       2441.14       5986.40  0.000000E+00       11190.8       42.4011       17758.8 -1.000000E+10 -1.000000E+10 -1.000000E+10 -1.000000E+10 -1.000000E+10  0.000000E+00  0.000000E+00  0.000000E+00  0.000000E+00  0.000000E+00  0.000000E+00  0.000000E+00  0.000000E+00  0.000000E+00  0.000000E+00  0.000000E+00  0.000000E+00       Funtag_D
      39  1.997130E+05  1.546084E+05       45104.7       61.5740      0.167400       1.07535      0.216114      0.230905    -0.0582441    -0.0827561    0.00645736      0.953916       Opttag_D
      39       70.8024       2.12931       1.00000  0.000000E+00       1.00000  0.000000E+00      0.836527       37.0480       1.00000       45.5516  0.000000E+00       4.78793  0.000000E+00       4.78793  0.000000E+00  0.000000E+00      -9.57586       71.3295       2.03201        1       Scatag_D
      39       1170.81       4958.85  0.000000E+00  0.000000E+00       2202.73  0.000000E+00       2403.20       5900.58  0.000000E+00       10984.4       38.0374       17446.1 -1.000000E+10 -1.000000E+10 -1.000000E+10 -1.000000E+10 -1.000000E+10  0.000000E+00  0.000000E+00  0.000000E+00  0.000000E+00  0.000000E+00  0.000000E+00  0.000000E+00  0.000000E+00  0.000000E+00  0.000000E+00  0.000000E+00  0.000000E+00       Funtag_D
      40  1.991961E+05  1.546450E+05       44551.1       34.4256      0.168330       1.08959      0.216130      0.230987    -0.0584561    -0.0831242    0.00634275      0.941622       Opttag_D
      40       70.8490       2.13796       1.00000  0.000000E+00       1.00000  0.000000E+00      0.836488       37.0745       1.00000       45.5576  0.000000E+00       4.78612  0.000000E+00       4.78612  0.000000E+00  0.000000E+00      -9.57223       71.3295       2.03201        1       Scatag_D
      40       1129.62       4831.86  0.000000E+00  0.000000E+00       2201.92  0.000000E+00       2383.20       5747.82  0.000000E+00       10929.6       39.3435       17287.7 -1.000000E+10 -1.000000E+10 -1.000000E+10 -1.000000E+10 -1.000000E+10  0.000000E+00  0.000000E+00  0.000000E+00  0.000000E+00  0.000000E+00  0.000000E+00  0.000000E+00  0.000000E+00  0.000000E+00  0.000000E+00  0.000000E+00  0.000000E+00       Funtag_D
      41  1.986753E+05  1.547027E+05       43972.7       35.6426      0.169758       1.11174      0.216155      0.231119    -0.0587895    -0.0837149    0.00625177      0.940964       Opttag_D
      41       70.9219       2.15165       1.00000  0.000000E+00       1.00000  0.000000E+00      0.836435       37.1185       1.00000       45.5634  0.000000E+00       4.78382  0.000000E+00       4.78382  0.000000E+00  0.000000E+00      -9.56763       71.3295       2.03201        1       Scatag_D
      41       1097.45       4825.11  0.000000E+00  0.000000E+00       2200.93  0.000000E+00       2349.41       5580.39  0.000000E+00       10841.5       43.6003       17034.3 -1.000000E+10 -1.000000E+10 -1.000000E+10 -1.000000E+10 -1.000000E+10  0.000000E+00  0.000000E+00  0.000000E+00  0.000000E+00  0.000000E+00  0.000000E+00  0.000000E+00  0.000000E+00  0.000000E+00  0.000000E+00  0.000000E+00  0.000000E+00       Funtag_D
      42  1.981851E+05  1.547972E+05       43387.9       39.4542      0.172356       1.14927      0.216191      0.231355    -0.0593363    -0.0847251    0.00624050      0.940332       Opttag_D
      42       71.0510       2.17509       1.00000  0.000000E+00       1.00000  0.000000E+00      0.836350       37.1927       1.00000       45.5890  0.000000E+00       4.77874  0.000000E+00       4.77874  0.000000E+00  0.000000E+00      -9.55749       71.3295       2.03201        1       Scatag_D
      42       1093.49       4818.63  0.000000E+00  0.000000E+00       2200.04  0.000000E+00       2313.64       5439.98  0.000000E+00       10704.1       46.4341       16771.6 -1.000000E+10 -1.000000E+10 -1.000000E+10 -1.000000E+10 -1.000000E+10  0.000000E+00  0.000000E+00  0.000000E+00  0.000000E+00  0.000000E+00  0.000000E+00  0.000000E+00  0.000000E+00  0.000000E+00  0.000000E+00  0.000000E+00  0.000000E+00       Funtag_D
      43  1.978143E+05  1.550071E+05       42807.2       80.9152      0.178225       1.22977      0.216279      0.231855    -0.0605503    -0.0869072    0.00636028      0.958457       Opttag_D
      43       71.3416       2.22768       1.00000  0.000000E+00       1.00000  0.000000E+00      0.836200       37.3537       1.00000       45.6658  0.000000E+00       4.76703  0.000000E+00       4.76703  0.000000E+00  0.000000E+00      -9.53406       71.3295       2.03201        1       Scatag_D
      43       1135.87       5006.18  0.000000E+00  0.000000E+00       2199.10  0.000000E+00       2259.06       5291.07  0.000000E+00       10429.7       48.7007       16437.6 -1.000000E+10 -1.000000E+10 -1.000000E+10 -1.000000E+10 -1.000000E+10  0.000000E+00  0.000000E+00  0.000000E+00  0.000000E+00  0.000000E+00  0.000000E+00  0.000000E+00  0.000000E+00  0.000000E+00  0.000000E+00  0.000000E+00  0.000000E+00       Funtag_D
      44  1.972190E+05  1.550346E+05       42184.4       30.5439      0.179222       1.24280      0.216285      0.231934    -0.0607097    -0.0872273    0.00618509      0.933785       Opttag_D
      44       71.3948       2.23631       1.00000  0.000000E+00       1.00000  0.000000E+00      0.836174       37.3807       1.00000       45.6893  0.000000E+00       4.76421  0.000000E+00       4.76421  0.000000E+00  0.000000E+00      -9.52841       71.3295       2.03201        1       Scatag_D
      44       1074.16       4751.76  0.000000E+00  0.000000E+00       2198.87  0.000000E+00       2255.33       5123.88  0.000000E+00       10384.4       45.6686       16350.3 -1.000000E+10 -1.000000E+10 -1.000000E+10 -1.000000E+10 -1.000000E+10  0.000000E+00  0.000000E+00  0.000000E+00  0.000000E+00  0.000000E+00  0.000000E+00  0.000000E+00  0.000000E+00  0.000000E+00  0.000000E+00  0.000000E+00  0.000000E+00       Funtag_D
      45  1.969222E+05  1.550495E+05       41872.7       28.4663      0.180045       1.25380      0.216270      0.231983    -0.0607957    -0.0874181    0.00612335      0.931494       Opttag_D
      45       71.4440       2.24342       1.00000  0.000000E+00       1.00000  0.000000E+00      0.836152       37.4053       1.00000       45.7128  0.000000E+00       4.76138  0.000000E+00       4.76138  0.000000E+00  0.000000E+00      -9.52275       71.3295       2.03201        1       Scatag_D
      45       1052.82       4728.48  0.000000E+00  0.000000E+00       2198.23  0.000000E+00       2252.66       5027.02  0.000000E+00       10341.3       42.3812       16229.9 -1.000000E+10 -1.000000E+10 -1.000000E+10 -1.000000E+10 -1.000000E+10  0.000000E+00  0.000000E+00  0.000000E+00  0.000000E+00  0.000000E+00  0.000000E+00  0.000000E+00  0.000000E+00  0.000000E+00  0.000000E+00  0.000000E+00  0.000000E+00       Funtag_D
      46  1.965158E+05  1.551213E+05       41394.5       34.0275      0.182507       1.28617      0.216278      0.232155    -0.0612112    -0.0881264    0.00608913      0.930230       Opttag_D
      46       71.5818       2.26611       1.00000  0.000000E+00       1.00000  0.000000E+00      0.836102       37.4767       1.00000       45.7679  0.000000E+00       4.75464  0.000000E+00       4.75464  0.000000E+00  0.000000E+00      -9.50929       71.3295       2.03201        1       Scatag_D
      46       1041.09       4715.65  0.000000E+00  0.000000E+00       2196.98  0.000000E+00       2237.82       4939.06  0.000000E+00       10227.2       39.2716       15997.4 -1.000000E+10 -1.000000E+10 -1.000000E+10 -1.000000E+10 -1.000000E+10  0.000000E+00  0.000000E+00  0.000000E+00  0.000000E+00  0.000000E+00  0.000000E+00  0.000000E+00  0.000000E+00  0.000000E+00  0.000000E+00  0.000000E+00  0.000000E+00       Funtag_D
      47  1.962347E+05  1.552836E+05       40951.1       88.8310      0.187314       1.34778      0.216334      0.232446    -0.0621500    -0.0894365    0.00621680      0.956015       Opttag_D
      47       71.8499       2.31264       1.00000  0.000000E+00       1.00000  0.000000E+00      0.836027       37.6164       1.00000       45.8737  0.000000E+00       4.74276  0.000000E+00       4.74276  0.000000E+00  0.000000E+00      -9.48551       71.3295       2.03201        1       Scatag_D
      47       1085.21       4980.70  0.000000E+00  0.000000E+00       2194.92  0.000000E+00       2202.65       4808.57  0.000000E+00       10019.7       38.3478       15621.0 -1.000000E+10 -1.000000E+10 -1.000000E+10 -1.000000E+10 -1.000000E+10  0.000000E+00  0.000000E+00  0.000000E+00  0.000000E+00  0.000000E+00  0.000000E+00  0.000000E+00  0.000000E+00  0.000000E+00  0.000000E+00  0.000000E+00  0.000000E+00       Funtag_D
      48  1.956700E+05  1.553313E+05       40338.7       34.8603      0.188638       1.36443      0.216357      0.232534    -0.0624257    -0.0898492    0.00603663      0.926688       Opttag_D
      48       71.9229       2.32565       1.00000  0.000000E+00       1.00000  0.000000E+00      0.836008       37.6541       1.00000       45.9025  0.000000E+00       4.73967  0.000000E+00       4.73967  0.000000E+00  0.000000E+00      -9.47934       71.3295       2.03201        1       Scatag_D
      48       1023.22       4679.81  0.000000E+00  0.000000E+00       2194.49  0.000000E+00       2192.22       4712.73  0.000000E+00       9964.09       38.6290       15533.5 -1.000000E+10 -1.000000E+10 -1.000000E+10 -1.000000E+10 -1.000000E+10  0.000000E+00  0.000000E+00  0.000000E+00  0.000000E+00  0.000000E+00  0.000000E+00  0.000000E+00  0.000000E+00  0.000000E+00  0.000000E+00  0.000000E+00  0.000000E+00       Funtag_D
      49  1.953661E+05  1.553626E+05       40003.5       23.9856      0.189493       1.37562      0.216368      0.232575    -0.0626065    -0.0900781    0.00595886      0.923779       Opttag_D
      49       71.9731       2.33456       1.00000  0.000000E+00       1.00000  0.000000E+00      0.835991       37.6816       1.00000       45.9197  0.000000E+00       4.73806  0.000000E+00       4.73806  0.000000E+00  0.000000E+00      -9.47612       71.3295       2.03201        1       Scatag_D
      49       997.022       4650.48  0.000000E+00  0.000000E+00       2193.81  0.000000E+00       2177.03       4592.35  0.000000E+00       9919.61       40.5764       15432.7 -1.000000E+10 -1.000000E+10 -1.000000E+10 -1.000000E+10 -1.000000E+10  0.000000E+00  0.000000E+00  0.000000E+00  0.000000E+00  0.000000E+00  0.000000E+00  0.000000E+00  0.000000E+00  0.000000E+00  0.000000E+00  0.000000E+00  0.000000E+00       Funtag_D
      50  1.950698E+05  1.554238E+05       39646.0       29.2035      0.191337       1.39884      0.216391      0.232673    -0.0629605    -0.0905519    0.00592738      0.923460       Opttag_D
      50       72.0783       2.35278       1.00000  0.000000E+00       1.00000  0.000000E+00      0.835951       37.7376       1.00000       45.9621  0.000000E+00       4.73414  0.000000E+00       4.73414  0.000000E+00  0.000000E+00      -9.46827       71.3295       2.03201        1       Scatag_D
      50       986.515       4647.26  0.000000E+00  0.000000E+00       2192.85  0.000000E+00       2155.14       4485.46  0.000000E+00       9836.47       43.0074       15299.3 -1.000000E+10 -1.000000E+10 -1.000000E+10 -1.000000E+10 -1.000000E+10  0.000000E+00  0.000000E+00  0.000000E+00  0.000000E+00  0.000000E+00  0.000000E+00  0.000000E+00  0.000000E+00  0.000000E+00  0.000000E+00  0.000000E+00  0.000000E+00       Funtag_D
      51  1.948373E+05  1.554820E+05       39355.3       62.3569      0.193880       1.43012      0.216385      0.232807    -0.0632973    -0.0910668    0.00593749      0.929195       Opttag_D
      51       72.2274       2.37590       1.00000  0.000000E+00       1.00000  0.000000E+00      0.835869       37.8141       1.00000       46.0354  0.000000E+00       4.72801  0.000000E+00       4.72801  0.000000E+00  0.000000E+00      -9.45602       71.3295       2.03201        1       Scatag_D
      51       989.882       4705.17  0.000000E+00  0.000000E+00       2191.20  0.000000E+00       2131.55       4429.58  0.000000E+00       9723.57       42.7661       15141.6 -1.000000E+10 -1.000000E+10 -1.000000E+10 -1.000000E+10 -1.000000E+10  0.000000E+00  0.000000E+00  0.000000E+00  0.000000E+00  0.000000E+00  0.000000E+00  0.000000E+00  0.000000E+00  0.000000E+00  0.000000E+00  0.000000E+00  0.000000E+00       Funtag_D
      52  1.945062E+05  1.555136E+05       38992.6       26.2738      0.195238       1.44620      0.216384      0.232892    -0.0634800    -0.0913669    0.00587474      0.916434       Opttag_D
      52       72.3058       2.38836       1.00000  0.000000E+00       1.00000  0.000000E+00      0.835830       37.8546       1.00000       46.0744  0.000000E+00       4.72449  0.000000E+00       4.72449  0.000000E+00  0.000000E+00      -9.44898       71.3295       2.03201        1       Scatag_D
      52       969.068       4576.82  0.000000E+00  0.000000E+00       2190.65  0.000000E+00       2123.33       4318.70  0.000000E+00       9670.82       42.5552       15100.7 -1.000000E+10 -1.000000E+10 -1.000000E+10 -1.000000E+10 -1.000000E+10  0.000000E+00  0.000000E+00  0.000000E+00  0.000000E+00  0.000000E+00  0.000000E+00  0.000000E+00  0.000000E+00  0.000000E+00  0.000000E+00  0.000000E+00  0.000000E+00       Funtag_D
      53  1.943063E+05  1.555137E+05       38792.6       20.5070      0.195958       1.45460      0.216357      0.232925    -0.0634807    -0.0914470    0.00583796      0.912321       Opttag_D
      53       72.3529       2.39424       1.00000  0.000000E+00       1.00000  0.000000E+00      0.835795       37.8798       1.00000       46.1002  0.000000E+00       4.72186  0.000000E+00       4.72186  0.000000E+00  0.000000E+00      -9.44373       71.3295       2.03201        1       Scatag_D
      53       956.973       4535.83  0.000000E+00  0.000000E+00       2189.87  0.000000E+00       2121.26       4277.13  0.000000E+00       9639.73       41.1991       15030.6 -1.000000E+10 -1.000000E+10 -1.000000E+10 -1.000000E+10 -1.000000E+10  0.000000E+00  0.000000E+00  0.000000E+00  0.000000E+00  0.000000E+00  0.000000E+00  0.000000E+00  0.000000E+00  0.000000E+00  0.000000E+00  0.000000E+00  0.000000E+00       Funtag_D
      54  1.940650E+05  1.555209E+05       38544.1       27.7936      0.197290       1.46975      0.216315      0.232980    -0.0635224    -0.0916041    0.00581212      0.909704       Opttag_D
      54       72.4402       2.40596       1.00000  0.000000E+00       1.00000  0.000000E+00      0.835744       37.9285       1.00000       46.1436  0.000000E+00       4.71727  0.000000E+00       4.71727  0.000000E+00  0.000000E+00      -9.43453       71.3295       2.03201        1       Scatag_D
      54       948.520       4509.85  0.000000E+00  0.000000E+00       2188.62  0.000000E+00       2118.21       4224.25  0.000000E+00       9584.46       39.9663       14930.2 -1.000000E+10 -1.000000E+10 -1.000000E+10 -1.000000E+10 -1.000000E+10  0.000000E+00  0.000000E+00  0.000000E+00  0.000000E+00  0.000000E+00  0.000000E+00  0.000000E+00  0.000000E+00  0.000000E+00  0.000000E+00  0.000000E+00  0.000000E+00       Funtag_D
      55  1.937774E+05  1.555560E+05       38221.4       47.6588      0.200019       1.49950      0.216249      0.233081    -0.0637252    -0.0920395    0.00579398      0.908268       Opttag_D
      55       72.6151       2.43187       1.00000  0.000000E+00       1.00000  0.000000E+00      0.835679       38.0324       1.00000       46.2180  0.000000E+00       4.70856  0.000000E+00       4.70856  0.000000E+00  0.000000E+00      -9.41711       71.3295       2.03201        1       Scatag_D
      55       942.608       4495.62  0.000000E+00  0.000000E+00       2186.75  0.000000E+00       2111.88       4167.13  0.000000E+00       9479.53       39.5064       14798.4 -1.000000E+10 -1.000000E+10 -1.000000E+10 -1.000000E+10 -1.000000E+10  0.000000E+00  0.000000E+00  0.000000E+00  0.000000E+00  0.000000E+00  0.000000E+00  0.000000E+00  0.000000E+00  0.000000E+00  0.000000E+00  0.000000E+00  0.000000E+00       Funtag_D

 An atom has moved more than     1.50 A from the initial/last
 position - if this was an accepted trial point, probably need to update the
 non-bonded contact list.

      56  1.934872E+05  1.555937E+05       37893.5       27.7586      0.202301       1.52319      0.216210      0.233153    -0.0639433    -0.0923593    0.00577907      0.902818       Opttag_D
      56       72.7582       2.45412       1.00000  0.000000E+00       1.00000  0.000000E+00      0.835641       38.1185       1.00000       46.2783  0.000000E+00       4.70170  0.000000E+00       4.70170  0.000000E+00  0.000000E+00      -9.40341       71.3295       2.03201        1       Scatag_D
      56       937.763       4441.82  0.000000E+00  0.000000E+00       2185.72  0.000000E+00       2108.61       4036.17  0.000000E+00       9398.29       39.7034       14745.4 -1.000000E+10 -1.000000E+10 -1.000000E+10 -1.000000E+10 -1.000000E+10  0.000000E+00  0.000000E+00  0.000000E+00  0.000000E+00  0.000000E+00  0.000000E+00  0.000000E+00  0.000000E+00  0.000000E+00  0.000000E+00  0.000000E+00  0.000000E+00       Funtag_D

 An atom has moved more than     1.50 A from the initial/last
 position - need to update the non-bonded contact list.


 ############################################################################################################
 ###################################################### setting up contact terms
 ############################################################################################################

      57  1.933017E+05  1.556025E+05       37699.2       19.0506      0.202780       1.52794      0.216196      0.233154    -0.0639943    -0.0924389    0.00573001      0.900962       Opttag_D
      57       72.7907       2.45962       1.00000  0.000000E+00       1.00000  0.000000E+00      0.835640       38.1436       1.00000       46.2832  0.000000E+00       4.70027  0.000000E+00       4.70027  0.000000E+00  0.000000E+00      -9.40054       71.3295       2.03201        1       Scatag_D
      57       921.911       4423.58  0.000000E+00  0.000000E+00       2185.14  0.000000E+00       2100.53       3978.51  0.000000E+00       9374.46       40.3092       14674.7 -1.000000E+10 -1.000000E+10 -1.000000E+10 -1.000000E+10 -1.000000E+10  0.000000E+00  0.000000E+00  0.000000E+00  0.000000E+00  0.000000E+00  0.000000E+00  0.000000E+00  0.000000E+00  0.000000E+00  0.000000E+00  0.000000E+00  0.000000E+00       Funtag_D
      58  1.930404E+05  1.556255E+05       37415.0       25.2272      0.204083       1.54059      0.216163      0.233186    -0.0641271    -0.0926649    0.00569776      0.901286       Opttag_D
      58       72.8760       2.47369       1.00000  0.000000E+00       1.00000  0.000000E+00      0.835635       38.2038       1.00000       46.3019  0.000000E+00       4.69681  0.000000E+00       4.69681  0.000000E+00  0.000000E+00      -9.39361       71.3295       2.03201        1       Scatag_D
      58       911.560       4426.77  0.000000E+00  0.000000E+00       2183.83  0.000000E+00       2081.94       3920.59  0.000000E+00       9315.11       41.5252       14533.7 -1.000000E+10 -1.000000E+10 -1.000000E+10 -1.000000E+10 -1.000000E+10  0.000000E+00  0.000000E+00  0.000000E+00  0.000000E+00  0.000000E+00  0.000000E+00  0.000000E+00  0.000000E+00  0.000000E+00  0.000000E+00  0.000000E+00  0.000000E+00       Funtag_D
      59  1.928679E+05  1.556532E+05       37214.6       35.2636      0.206081       1.55957      0.216113      0.233225    -0.0642879    -0.0930306    0.00571359      0.901193       Opttag_D
      59       73.0005       2.49321       1.00000  0.000000E+00       1.00000  0.000000E+00      0.835604       38.2849       1.00000       46.3453  0.000000E+00       4.69088  0.000000E+00       4.69088  0.000000E+00  0.000000E+00      -9.38176       71.3295       2.03201        1       Scatag_D
      59       916.634       4425.85  0.000000E+00  0.000000E+00       2182.47  0.000000E+00       2067.22       3891.85  0.000000E+00       9242.08       41.6022       14446.9 -1.000000E+10 -1.000000E+10 -1.000000E+10 -1.000000E+10 -1.000000E+10  0.000000E+00  0.000000E+00  0.000000E+00  0.000000E+00  0.000000E+00  0.000000E+00  0.000000E+00  0.000000E+00  0.000000E+00  0.000000E+00  0.000000E+00  0.000000E+00       Funtag_D
      60  1.926710E+05  1.556632E+05       37007.9       20.6525      0.207063       1.56864      0.216085      0.233255    -0.0643453    -0.0931788    0.00568691      0.898597       Opttag_D
      60       73.0605       2.50190       1.00000  0.000000E+00       1.00000  0.000000E+00      0.835584       38.3174       1.00000       46.3735  0.000000E+00       4.68839  0.000000E+00       4.68839  0.000000E+00  0.000000E+00      -9.37677       71.3295       2.03201        1       Scatag_D
      60       908.092       4400.39  0.000000E+00  0.000000E+00       2181.83  0.000000E+00       2060.90       3801.48  0.000000E+00       9208.28       40.9292       14406.0 -1.000000E+10 -1.000000E+10 -1.000000E+10 -1.000000E+10 -1.000000E+10  0.000000E+00  0.000000E+00  0.000000E+00  0.000000E+00  0.000000E+00  0.000000E+00  0.000000E+00  0.000000E+00  0.000000E+00  0.000000E+00  0.000000E+00  0.000000E+00       Funtag_D
      61  1.924575E+05  1.556799E+05       36777.6       18.0204      0.208826       1.58983      0.216042      0.233305    -0.0644419    -0.0934421    0.00567065      0.896393       Opttag_D
      61       73.1672       2.51714       1.00000  0.000000E+00       1.00000  0.000000E+00      0.835545       38.3732       1.00000       46.4276  0.000000E+00       4.68371  0.000000E+00       4.68371  0.000000E+00  0.000000E+00      -9.36742       71.3295       2.03201        1       Scatag_D
      61       902.907       4378.83  0.000000E+00  0.000000E+00       2180.64  0.000000E+00       2052.47       3727.95  0.000000E+00       9153.15       39.7985       14341.9 -1.000000E+10 -1.000000E+10 -1.000000E+10 -1.000000E+10 -1.000000E+10  0.000000E+00  0.000000E+00  0.000000E+00  0.000000E+00  0.000000E+00  0.000000E+00  0.000000E+00  0.000000E+00  0.000000E+00  0.000000E+00  0.000000E+00  0.000000E+00       Funtag_D
      62  1.923230E+05  1.556968E+05       36626.2       23.8499      0.210281       1.61150      0.216012      0.233342    -0.0645399    -0.0936674    0.00565893      0.895395       Opttag_D
      62       73.2566       2.53044       1.00000  0.000000E+00       1.00000  0.000000E+00      0.835518       38.4234       1.00000       46.4701  0.000000E+00       4.68006  0.000000E+00       4.68006  0.000000E+00  0.000000E+00      -9.36012       71.3295       2.03201        1       Scatag_D
      62       899.180       4369.08  0.000000E+00  0.000000E+00       2179.52  0.000000E+00       2046.46       3694.89  0.000000E+00       9110.95       39.8167       14286.3 -1.000000E+10 -1.000000E+10 -1.000000E+10 -1.000000E+10 -1.000000E+10  0.000000E+00  0.000000E+00  0.000000E+00  0.000000E+00  0.000000E+00  0.000000E+00  0.000000E+00  0.000000E+00  0.000000E+00  0.000000E+00  0.000000E+00  0.000000E+00       Funtag_D
      63  1.921316E+05  1.557211E+05       36410.6       19.1968      0.212226       1.63967      0.215976      0.233377    -0.0646802    -0.0939322    0.00564981      0.893263       Opttag_D
      63       73.3761       2.54847       1.00000  0.000000E+00       1.00000  0.000000E+00      0.835484       38.4910       1.00000       46.5262  0.000000E+00       4.67472  0.000000E+00       4.67472  0.000000E+00  0.000000E+00      -9.34943       71.3295       2.03201        1       Scatag_D
      63       896.284       4348.31  0.000000E+00  0.000000E+00       2178.08  0.000000E+00       2040.36       3628.90  0.000000E+00       9059.80       40.1492       14218.7 -1.000000E+10 -1.000000E+10 -1.000000E+10 -1.000000E+10 -1.000000E+10  0.000000E+00  0.000000E+00  0.000000E+00  0.000000E+00  0.000000E+00  0.000000E+00  0.000000E+00  0.000000E+00  0.000000E+00  0.000000E+00  0.000000E+00  0.000000E+00       Funtag_D
      64  1.919418E+05  1.557513E+05       36190.6       32.0200      0.214643       1.67326      0.215931      0.233400    -0.0648548    -0.0943003    0.00562674      0.893019       Opttag_D
      64       73.5258       2.57124       1.00000  0.000000E+00       1.00000  0.000000E+00      0.835427       38.5793       1.00000       46.5994  0.000000E+00       4.66771  0.000000E+00       4.66771  0.000000E+00  0.000000E+00      -9.33542       71.3295       2.03201        1       Scatag_D
      64       888.978       4345.92  0.000000E+00  0.000000E+00       2175.68  0.000000E+00       2035.40       3568.37  0.000000E+00       9002.07       40.2120       14133.9 -1.000000E+10 -1.000000E+10 -1.000000E+10 -1.000000E+10 -1.000000E+10  0.000000E+00  0.000000E+00  0.000000E+00  0.000000E+00  0.000000E+00  0.000000E+00  0.000000E+00  0.000000E+00  0.000000E+00  0.000000E+00  0.000000E+00  0.000000E+00       Funtag_D
      65  1.917555E+05  1.557635E+05       35992.0       17.6881      0.216000       1.69120      0.215901      0.233394    -0.0649255    -0.0944045    0.00558865      0.890222       Opttag_D
      65       73.6109       2.58361       1.00000  0.000000E+00       1.00000  0.000000E+00      0.835375       38.6283       1.00000       46.6439  0.000000E+00       4.66328  0.000000E+00       4.66328  0.000000E+00  0.000000E+00      -9.32656       71.3295       2.03201        1       Scatag_D
      65       876.982       4318.75  0.000000E+00  0.000000E+00       2174.30  0.000000E+00       2033.76       3488.46  0.000000E+00       8972.14       40.0546       14087.6 -1.000000E+10 -1.000000E+10 -1.000000E+10 -1.000000E+10 -1.000000E+10  0.000000E+00  0.000000E+00  0.000000E+00  0.000000E+00  0.000000E+00  0.000000E+00  0.000000E+00  0.000000E+00  0.000000E+00  0.000000E+00  0.000000E+00  0.000000E+00       Funtag_D
      66  1.916379E+05  1.557565E+05       35881.4       15.8418      0.216252       1.69403      0.215879      0.233382    -0.0648854    -0.0943908    0.00555651      0.889232       Opttag_D
      66       73.6303       2.58542       1.00000  0.000000E+00       1.00000  0.000000E+00      0.835338       38.6389       1.00000       46.6577  0.000000E+00       4.66174  0.000000E+00       4.66174  0.000000E+00  0.000000E+00      -9.32348       71.3295       2.03201        1       Scatag_D
      66       866.924       4309.15  0.000000E+00  0.000000E+00       2173.47  0.000000E+00       2031.37       3457.47  0.000000E+00       8962.62       39.5252       14040.8 -1.000000E+10 -1.000000E+10 -1.000000E+10 -1.000000E+10 -1.000000E+10  0.000000E+00  0.000000E+00  0.000000E+00  0.000000E+00  0.000000E+00  0.000000E+00  0.000000E+00  0.000000E+00  0.000000E+00  0.000000E+00  0.000000E+00  0.000000E+00       Funtag_D
      67  1.914794E+05  1.557454E+05       35734.0       23.8671      0.217163       1.70386      0.215830      0.233383    -0.0648210    -0.0944211    0.00554357      0.888133       Opttag_D
      67       73.6919       2.59187       1.00000  0.000000E+00       1.00000  0.000000E+00      0.835241       38.6671       1.00000       46.7037  0.000000E+00       4.65754  0.000000E+00       4.65754  0.000000E+00  0.000000E+00      -9.31509       71.3295       2.03201        1       Scatag_D
      67       862.892       4298.50  0.000000E+00  0.000000E+00       2171.43  0.000000E+00       2026.10       3444.63  0.000000E+00       8934.60       38.5405       13957.3 -1.000000E+10 -1.000000E+10 -1.000000E+10 -1.000000E+10 -1.000000E+10  0.000000E+00  0.000000E+00  0.000000E+00  0.000000E+00  0.000000E+00  0.000000E+00  0.000000E+00  0.000000E+00  0.000000E+00  0.000000E+00  0.000000E+00  0.000000E+00       Funtag_D
      68  1.913428E+05  1.557543E+05       35588.5       26.0907      0.218781       1.72160      0.215790      0.233397    -0.0648722    -0.0946364    0.00554122      0.887304       Opttag_D
      68       73.7893       2.60469       1.00000  0.000000E+00       1.00000  0.000000E+00      0.835148       38.7105       1.00000       46.7738  0.000000E+00       4.65265  0.000000E+00       4.65265  0.000000E+00  0.000000E+00      -9.30531       71.3295       2.03201        1       Scatag_D
      68       862.162       4290.48  0.000000E+00  0.000000E+00       2170.09  0.000000E+00       2022.12       3398.81  0.000000E+00       8898.76       38.0483       13908.0 -1.000000E+10 -1.000000E+10 -1.000000E+10 -1.000000E+10 -1.000000E+10  0.000000E+00  0.000000E+00  0.000000E+00  0.000000E+00  0.000000E+00  0.000000E+00  0.000000E+00  0.000000E+00  0.000000E+00  0.000000E+00  0.000000E+00  0.000000E+00       Funtag_D
      69  1.912184E+05  1.557591E+05       35459.4       17.4142      0.219358       1.72738      0.215780      0.233411    -0.0648999    -0.0947275    0.00552859      0.885549       Opttag_D
      69       73.8222       2.60902       1.00000  0.000000E+00       1.00000  0.000000E+00      0.835120       38.7213       1.00000       46.7991  0.000000E+00       4.65108  0.000000E+00       4.65108  0.000000E+00  0.000000E+00      -9.30217       71.3295       2.03201        1       Scatag_D
      69       858.236       4273.53  0.000000E+00  0.000000E+00       2169.64  0.000000E+00       2018.99       3345.17  0.000000E+00       8883.51       38.0508       13872.3 -1.000000E+10 -1.000000E+10 -1.000000E+10 -1.000000E+10 -1.000000E+10  0.000000E+00  0.000000E+00  0.000000E+00  0.000000E+00  0.000000E+00  0.000000E+00  0.000000E+00  0.000000E+00  0.000000E+00  0.000000E+00  0.000000E+00  0.000000E+00       Funtag_D
      70  1.910461E+05  1.557867E+05       35259.4       15.6515      0.221303       1.74688      0.215763      0.233466    -0.0650596    -0.0950708    0.00553436      0.884015       Opttag_D
      70       73.9308       2.62476       1.00000  0.000000E+00       1.00000  0.000000E+00      0.835045       38.7627       1.00000       46.8769  0.000000E+00       4.64660  0.000000E+00       4.64660  0.000000E+00  0.000000E+00      -9.29320       71.3295       2.03201        1       Scatag_D
      70       860.028       4258.73  0.000000E+00  0.000000E+00       2168.37  0.000000E+00       2012.95       3285.69  0.000000E+00       8839.52       38.3118       13795.8 -1.000000E+10 -1.000000E+10 -1.000000E+10 -1.000000E+10 -1.000000E+10  0.000000E+00  0.000000E+00  0.000000E+00  0.000000E+00  0.000000E+00  0.000000E+00  0.000000E+00  0.000000E+00  0.000000E+00  0.000000E+00  0.000000E+00  0.000000E+00       Funtag_D
      71  1.909429E+05  1.558137E+05       35129.1       22.4586      0.223041       1.76296      0.215754      0.233514    -0.0652161    -0.0953366    0.00553404      0.883749       Opttag_D
      71       74.0270       2.63920       1.00000  0.000000E+00       1.00000  0.000000E+00      0.834972       38.8033       1.00000       46.9436  0.000000E+00       4.64242  0.000000E+00       4.64242  0.000000E+00  0.000000E+00      -9.28485       71.3295       2.03201        1       Scatag_D
      71       859.929       4256.17  0.000000E+00  0.000000E+00       2167.36  0.000000E+00       2009.52       3249.72  0.000000E+00       8802.44       38.1370       13745.9 -1.000000E+10 -1.000000E+10 -1.000000E+10 -1.000000E+10 -1.000000E+10  0.000000E+00  0.000000E+00  0.000000E+00  0.000000E+00  0.000000E+00  0.000000E+00  0.000000E+00  0.000000E+00  0.000000E+00  0.000000E+00  0.000000E+00  0.000000E+00       Funtag_D
      72  1.908156E+05  1.558441E+05       34971.5       16.6817      0.224652       1.77686      0.215751      0.233593    -0.0653919    -0.0956582    0.00551804      0.883223       Opttag_D
      72       74.1136       2.65278       1.00000  0.000000E+00       1.00000  0.000000E+00      0.834910       38.8400       1.00000       47.0028  0.000000E+00       4.63900  0.000000E+00       4.63900  0.000000E+00  0.000000E+00      -9.27800       71.3295       2.03201        1       Scatag_D
      72       854.964       4251.11  0.000000E+00  0.000000E+00       2166.32  0.000000E+00       2006.47       3187.63  0.000000E+00       8768.73       37.9239       13698.4 -1.000000E+10 -1.000000E+10 -1.000000E+10 -1.000000E+10 -1.000000E+10  0.000000E+00  0.000000E+00  0.000000E+00  0.000000E+00  0.000000E+00  0.000000E+00  0.000000E+00  0.000000E+00  0.000000E+00  0.000000E+00  0.000000E+00  0.000000E+00       Funtag_D
      73  1.906714E+05  1.558838E+05       34787.6       22.5658      0.226993       1.79467      0.215744      0.233730    -0.0656212    -0.0961200    0.00549743      0.884247       Opttag_D
      73       74.2352       2.67129       1.00000  0.000000E+00       1.00000  0.000000E+00      0.834792       38.8869       1.00000       47.0918  0.000000E+00       4.63398  0.000000E+00       4.63398  0.000000E+00  0.000000E+00      -9.26795       71.3295       2.03201        1       Scatag_D
      73       848.590       4260.97  0.000000E+00  0.000000E+00       2164.52  0.000000E+00       2000.73       3138.91  0.000000E+00       8720.39       37.2550       13616.2 -1.000000E+10 -1.000000E+10 -1.000000E+10 -1.000000E+10 -1.000000E+10  0.000000E+00  0.000000E+00  0.000000E+00  0.000000E+00  0.000000E+00  0.000000E+00  0.000000E+00  0.000000E+00  0.000000E+00  0.000000E+00  0.000000E+00  0.000000E+00       Funtag_D
      74  1.905643E+05  1.559085E+05       34655.8       18.8606      0.228454       1.80441      0.215741      0.233833    -0.0657643    -0.0964263    0.00549030      0.883705       Opttag_D
      74       74.3083       2.68241       1.00000  0.000000E+00       1.00000  0.000000E+00      0.834715       38.9146       1.00000       47.1486  0.000000E+00       4.63085  0.000000E+00       4.63085  0.000000E+00  0.000000E+00      -9.26170       71.3295       2.03201        1       Scatag_D
      74       846.388       4255.75  0.000000E+00  0.000000E+00       2163.59  0.000000E+00       1996.48       3105.33  0.000000E+00       8691.20       36.7045       13560.4 -1.000000E+10 -1.000000E+10 -1.000000E+10 -1.000000E+10 -1.000000E+10  0.000000E+00  0.000000E+00  0.000000E+00  0.000000E+00  0.000000E+00  0.000000E+00  0.000000E+00  0.000000E+00  0.000000E+00  0.000000E+00  0.000000E+00  0.000000E+00       Funtag_D
      75  1.904766E+05  1.559089E+05       34567.7       15.1659      0.228869       1.80600      0.215732      0.233875    -0.0657668    -0.0965088    0.00547864      0.883588       Opttag_D
      75       74.3272       2.68447       1.00000  0.000000E+00       1.00000  0.000000E+00      0.834673       38.9181       1.00000       47.1663  0.000000E+00       4.62966  0.000000E+00       4.62966  0.000000E+00  0.000000E+00      -9.25932       71.3295       2.03201        1       Scatag_D
      75       842.796       4254.62  0.000000E+00  0.000000E+00       2162.90  0.000000E+00       1993.35       3080.53  0.000000E+00       8679.58       36.4283       13517.5 -1.000000E+10 -1.000000E+10 -1.000000E+10 -1.000000E+10 -1.000000E+10  0.000000E+00  0.000000E+00  0.000000E+00  0.000000E+00  0.000000E+00  0.000000E+00  0.000000E+00  0.000000E+00  0.000000E+00  0.000000E+00  0.000000E+00  0.000000E+00       Funtag_D
      76  1.903092E+05  1.559113E+05       34397.9       17.5947      0.230169       1.81017      0.215710      0.234018    -0.0657804    -0.0967683    0.00548161      0.884354       Opttag_D
      76       74.3825       2.69024       1.00000  0.000000E+00       1.00000  0.000000E+00      0.834537       38.9237       1.00000       47.2231  0.000000E+00       4.62638  0.000000E+00       4.62638  0.000000E+00  0.000000E+00      -9.25275       71.3295       2.03201        1       Scatag_D
      76       843.712       4262.00  0.000000E+00  0.000000E+00       2161.22  0.000000E+00       1986.37       3059.21  0.000000E+00       8645.96       36.0506       13403.4 -1.000000E+10 -1.000000E+10 -1.000000E+10 -1.000000E+10 -1.000000E+10  0.000000E+00  0.000000E+00  0.000000E+00  0.000000E+00  0.000000E+00  0.000000E+00  0.000000E+00  0.000000E+00  0.000000E+00  0.000000E+00  0.000000E+00  0.000000E+00       Funtag_D
      77  1.902212E+05  1.559467E+05       34274.5       33.6240      0.232388       1.82139      0.215716      0.234201    -0.0659854    -0.0972323    0.00549354      0.886637       Opttag_D
      77       74.4782       2.70413       1.00000  0.000000E+00       1.00000  0.000000E+00      0.834394       38.9432       1.00000       47.3109  0.000000E+00       4.62199  0.000000E+00       4.62199  0.000000E+00  0.000000E+00      -9.24398       71.3295       2.03201        1       Scatag_D
      77       847.386       4284.04  0.000000E+00  0.000000E+00       2160.88  0.000000E+00       1983.34       3047.71  0.000000E+00       8605.95       35.7805       13309.4 -1.000000E+10 -1.000000E+10 -1.000000E+10 -1.000000E+10 -1.000000E+10  0.000000E+00  0.000000E+00  0.000000E+00  0.000000E+00  0.000000E+00  0.000000E+00  0.000000E+00  0.000000E+00  0.000000E+00  0.000000E+00  0.000000E+00  0.000000E+00       Funtag_D
      78  1.901012E+05  1.559526E+05       34148.6       18.5504      0.232677       1.82261      0.215719      0.234239    -0.0660195    -0.0973249    0.00547437      0.885709       Opttag_D
      78       74.4886       2.70563       1.00000  0.000000E+00       1.00000  0.000000E+00      0.834373       38.9419       1.00000       47.3217  0.000000E+00       4.62161  0.000000E+00       4.62161  0.000000E+00  0.000000E+00      -9.24322       71.3295       2.03201        1       Scatag_D
      78       841.484       4275.07  0.000000E+00  0.000000E+00       2160.77  0.000000E+00       1982.28       2979.77  0.000000E+00       8599.53       35.8371       13273.9 -1.000000E+10 -1.000000E+10 -1.000000E+10 -1.000000E+10 -1.000000E+10  0.000000E+00  0.000000E+00  0.000000E+00  0.000000E+00  0.000000E+00  0.000000E+00  0.000000E+00  0.000000E+00  0.000000E+00  0.000000E+00  0.000000E+00  0.000000E+00       Funtag_D
      79  1.899612E+05  1.559878E+05       33973.4       14.7142      0.234277       1.83075      0.215741      0.234385    -0.0662229    -0.0977315    0.00546553      0.886163       Opttag_D
      79       74.5527       2.71572       1.00000  0.000000E+00       1.00000  0.000000E+00      0.834277       38.9493       1.00000       47.3835  0.000000E+00       4.61919  0.000000E+00       4.61919  0.000000E+00  0.000000E+00      -9.23837       71.3295       2.03201        1       Scatag_D
      79       838.769       4279.45  0.000000E+00  0.000000E+00       2160.87  0.000000E+00       1979.60       2934.69  0.000000E+00       8572.77       35.8900       13171.4 -1.000000E+10 -1.000000E+10 -1.000000E+10 -1.000000E+10 -1.000000E+10  0.000000E+00  0.000000E+00  0.000000E+00  0.000000E+00  0.000000E+00  0.000000E+00  0.000000E+00  0.000000E+00  0.000000E+00  0.000000E+00  0.000000E+00  0.000000E+00       Funtag_D
      80  1.898676E+05  1.560183E+05       33849.3       16.8814      0.235623       1.83749      0.215768      0.234505    -0.0663995    -0.0980617    0.00546116      0.886451       Opttag_D
      80       74.6060       2.72412       1.00000  0.000000E+00       1.00000  0.000000E+00      0.834190       38.9543       1.00000       47.4381  0.000000E+00       4.61719  0.000000E+00       4.61719  0.000000E+00  0.000000E+00      -9.23439       71.3295       2.03201        1       Scatag_D
      80       837.428       4282.23  0.000000E+00  0.000000E+00       2160.96  0.000000E+00       1976.41       2913.38  0.000000E+00       8551.77       35.7105       13091.4 -1.000000E+10 -1.000000E+10 -1.000000E+10 -1.000000E+10 -1.000000E+10  0.000000E+00  0.000000E+00  0.000000E+00  0.000000E+00  0.000000E+00  0.000000E+00  0.000000E+00  0.000000E+00  0.000000E+00  0.000000E+00  0.000000E+00  0.000000E+00       Funtag_D
      81  1.896867E+05  1.561088E+05       33577.9       33.7266      0.239259       1.85505      0.215856      0.234868    -0.0669230    -0.0990159    0.00546115      0.884913       Opttag_D
      81       74.7424       2.74638       1.00000  0.000000E+00       1.00000  0.000000E+00      0.833947       38.9589       1.00000       47.5848  0.000000E+00       4.61198  0.000000E+00       4.61198  0.000000E+00  0.000000E+00      -9.22397       71.3295       2.03201        1       Scatag_D
      81       837.426       4267.39  0.000000E+00  0.000000E+00       2160.56  0.000000E+00       1964.12       2879.42  0.000000E+00       8500.99       35.0998       12932.9 -1.000000E+10 -1.000000E+10 -1.000000E+10 -1.000000E+10 -1.000000E+10  0.000000E+00  0.000000E+00  0.000000E+00  0.000000E+00  0.000000E+00  0.000000E+00  0.000000E+00  0.000000E+00  0.000000E+00  0.000000E+00  0.000000E+00  0.000000E+00       Funtag_D
      82  1.895802E+05  1.561569E+05       33423.3       34.4865      0.241061       1.86348      0.215908      0.235050    -0.0672010    -0.0994624    0.00545742      0.878708       Opttag_D
      82       74.8051       2.75659       1.00000  0.000000E+00       1.00000  0.000000E+00      0.833808       38.9516       1.00000       47.6638  0.000000E+00       4.61048  0.000000E+00       4.61048  0.000000E+00  0.000000E+00      -9.22096       71.3295       2.03201        1       Scatag_D
      82       836.280       4207.76  0.000000E+00  0.000000E+00       2159.78  0.000000E+00       1955.09       2856.85  0.000000E+00       8477.06       34.7404       12895.8 -1.000000E+10 -1.000000E+10 -1.000000E+10 -1.000000E+10 -1.000000E+10  0.000000E+00  0.000000E+00  0.000000E+00  0.000000E+00  0.000000E+00  0.000000E+00  0.000000E+00  0.000000E+00  0.000000E+00  0.000000E+00  0.000000E+00  0.000000E+00       Funtag_D
      83  1.894387E+05  1.561215E+05       33317.2       14.7233      0.239798       1.85760      0.215875      0.234940    -0.0669963    -0.0991390    0.00542392      0.877668       Opttag_D
      83       74.7571       2.74802       1.00000  0.000000E+00       1.00000  0.000000E+00      0.833870       38.9453       1.00000       47.6142  0.000000E+00       4.61222  0.000000E+00       4.61222  0.000000E+00  0.000000E+00      -9.22443       71.3295       2.03201        1       Scatag_D
      83       826.045       4197.80  0.000000E+00  0.000000E+00       2159.09  0.000000E+00       1953.82       2796.11  0.000000E+00       8487.17       34.7934       12862.4 -1.000000E+10 -1.000000E+10 -1.000000E+10 -1.000000E+10 -1.000000E+10  0.000000E+00  0.000000E+00  0.000000E+00  0.000000E+00  0.000000E+00  0.000000E+00  0.000000E+00  0.000000E+00  0.000000E+00  0.000000E+00  0.000000E+00  0.000000E+00       Funtag_D
      84  1.893575E+05  1.561182E+05       33239.3       11.4942      0.239758       1.85742      0.215872      0.234952    -0.0669774    -0.0991468    0.00541945      0.874860       Opttag_D
      84       74.7518       2.74670       1.00000  0.000000E+00       1.00000  0.000000E+00      0.833857       38.9385       1.00000       47.6116  0.000000E+00       4.61252  0.000000E+00       4.61252  0.000000E+00  0.000000E+00      -9.22504       71.3295       2.03201        1       Scatag_D
      84       824.686       4170.98  0.000000E+00  0.000000E+00       2158.12  0.000000E+00       1950.12       2782.54  0.000000E+00       8483.50       34.8278       12834.5 -1.000000E+10 -1.000000E+10 -1.000000E+10 -1.000000E+10 -1.000000E+10  0.000000E+00  0.000000E+00  0.000000E+00  0.000000E+00  0.000000E+00  0.000000E+00  0.000000E+00  0.000000E+00  0.000000E+00  0.000000E+00  0.000000E+00  0.000000E+00       Funtag_D
      85  1.892639E+05  1.561224E+05       33141.5       14.7087      0.240095       1.85901      0.215882      0.235013    -0.0670015    -0.0992684    0.00543190      0.870455       Opttag_D
      85       74.7571       2.74685       1.00000  0.000000E+00       1.00000  0.000000E+00      0.833815       38.9285       1.00000       47.6217  0.000000E+00       4.61270  0.000000E+00       4.61270  0.000000E+00  0.000000E+00      -9.22540       71.3295       2.03201        1       Scatag_D
      85       828.480       4129.09  0.000000E+00  0.000000E+00       2156.49  0.000000E+00       1945.10       2773.97  0.000000E+00       8473.52       34.9089       12799.9 -1.000000E+10 -1.000000E+10 -1.000000E+10 -1.000000E+10 -1.000000E+10  0.000000E+00  0.000000E+00  0.000000E+00  0.000000E+00  0.000000E+00  0.000000E+00  0.000000E+00  0.000000E+00  0.000000E+00  0.000000E+00  0.000000E+00  0.000000E+00       Funtag_D
      86  1.891908E+05  1.561467E+05       33044.0       33.2858      0.241558       1.86566      0.215915      0.235184    -0.0671423    -0.0996888    0.00545615      0.864950       Opttag_D
      86       74.7956       2.75181       1.00000  0.000000E+00       1.00000  0.000000E+00      0.833701       38.9124       1.00000       47.6687  0.000000E+00       4.61191  0.000000E+00       4.61191  0.000000E+00  0.000000E+00      -9.22382       71.3295       2.03201        1       Scatag_D
      86       835.892       4077.03  0.000000E+00  0.000000E+00       2153.77  0.000000E+00       1939.46       2799.61  0.000000E+00       8446.92       34.9111       12756.4 -1.000000E+10 -1.000000E+10 -1.000000E+10 -1.000000E+10 -1.000000E+10  0.000000E+00  0.000000E+00  0.000000E+00  0.000000E+00  0.000000E+00  0.000000E+00  0.000000E+00  0.000000E+00  0.000000E+00  0.000000E+00  0.000000E+00  0.000000E+00       Funtag_D
      87  1.890739E+05  1.561456E+05       32928.3       13.7215      0.241707       1.86628      0.215914      0.235211    -0.0671358    -0.0997570    0.00545004      0.863814       Opttag_D
      87       74.7963       2.75142       1.00000  0.000000E+00       1.00000  0.000000E+00      0.833688       38.9091       1.00000       47.6700  0.000000E+00       4.61225  0.000000E+00       4.61225  0.000000E+00  0.000000E+00      -9.22450       71.3295       2.03201        1       Scatag_D
      87       834.020       4066.32  0.000000E+00  0.000000E+00       2152.99  0.000000E+00       1938.69       2725.64  0.000000E+00       8441.30       34.9685       12734.3 -1.000000E+10 -1.000000E+10 -1.000000E+10 -1.000000E+10 -1.000000E+10  0.000000E+00  0.000000E+00  0.000000E+00  0.000000E+00  0.000000E+00  0.000000E+00  0.000000E+00  0.000000E+00  0.000000E+00  0.000000E+00  0.000000E+00  0.000000E+00       Funtag_D
      88  1.890085E+05  1.561424E+05       32866.1       10.2996      0.241827       1.86673      0.215910      0.235221    -0.0671175    -0.0997942    0.00541583      0.863047       Opttag_D
      88       74.7978       2.75115       1.00000  0.000000E+00       1.00000  0.000000E+00      0.833680       38.9082       1.00000       47.6713  0.000000E+00       4.61244  0.000000E+00       4.61244  0.000000E+00  0.000000E+00      -9.22488       71.3295       2.03201        1       Scatag_D
      88       823.583       4059.10  0.000000E+00  0.000000E+00       2152.66  0.000000E+00       1938.39       2719.59  0.000000E+00       8435.63       34.9382       12702.2 -1.000000E+10 -1.000000E+10 -1.000000E+10 -1.000000E+10 -1.000000E+10  0.000000E+00  0.000000E+00  0.000000E+00  0.000000E+00  0.000000E+00  0.000000E+00  0.000000E+00  0.000000E+00  0.000000E+00  0.000000E+00  0.000000E+00  0.000000E+00       Funtag_D
      89  1.889461E+05  1.561453E+05       32800.9       12.6958      0.242317       1.86866      0.215911      0.235275    -0.0671338    -0.0999312    0.00539642      0.862247       Opttag_D
      89       74.8086       2.75213       1.00000  0.000000E+00       1.00000  0.000000E+00      0.833645       38.9041       1.00000       47.6845  0.000000E+00       4.61242  0.000000E+00       4.61242  0.000000E+00  0.000000E+00      -9.22484       71.3295       2.03201        1       Scatag_D
      89       817.691       4051.58  0.000000E+00  0.000000E+00       2151.78  0.000000E+00       1937.03       2718.91  0.000000E+00       8423.28       34.8717       12665.7 -1.000000E+10 -1.000000E+10 -1.000000E+10 -1.000000E+10 -1.000000E+10  0.000000E+00  0.000000E+00  0.000000E+00  0.000000E+00  0.000000E+00  0.000000E+00  0.000000E+00  0.000000E+00  0.000000E+00  0.000000E+00  0.000000E+00  0.000000E+00       Funtag_D
      90  1.888357E+05  1.561503E+05       32685.4       25.4601      0.243246       1.87167      0.215915      0.235409    -0.0671632     -0.100225    0.00537449      0.860756       Opttag_D
      90       74.8252       2.75333       1.00000  0.000000E+00       1.00000  0.000000E+00      0.833572       38.8930       1.00000       47.7093  0.000000E+00       4.61251  0.000000E+00       4.61251  0.000000E+00  0.000000E+00      -9.22502       71.3295       2.03201        1       Scatag_D
      90       811.057       4037.58  0.000000E+00  0.000000E+00       2149.82  0.000000E+00       1931.58       2732.28  0.000000E+00       8396.89       34.6735       12591.5 -1.000000E+10 -1.000000E+10 -1.000000E+10 -1.000000E+10 -1.000000E+10  0.000000E+00  0.000000E+00  0.000000E+00  0.000000E+00  0.000000E+00  0.000000E+00  0.000000E+00  0.000000E+00  0.000000E+00  0.000000E+00  0.000000E+00  0.000000E+00       Funtag_D
      91  1.887139E+05  1.561645E+05       32549.4       14.3936      0.244541       1.87581      0.215931      0.235558    -0.0672448     -0.100566    0.00538591      0.861319       Opttag_D
      91       74.8492       2.75599       1.00000  0.000000E+00       1.00000  0.000000E+00      0.833476       38.8794       1.00000       47.7456  0.000000E+00       4.61233  0.000000E+00       4.61233  0.000000E+00  0.000000E+00      -9.22467       71.3295       2.03201        1       Scatag_D
      91       814.510       4042.86  0.000000E+00  0.000000E+00       2147.78  0.000000E+00       1926.76       2673.21  0.000000E+00       8370.70       34.5435       12539.0 -1.000000E+10 -1.000000E+10 -1.000000E+10 -1.000000E+10 -1.000000E+10  0.000000E+00  0.000000E+00  0.000000E+00  0.000000E+00  0.000000E+00  0.000000E+00  0.000000E+00  0.000000E+00  0.000000E+00  0.000000E+00  0.000000E+00  0.000000E+00       Funtag_D
      92  1.886375E+05  1.561702E+05       32467.2       11.7094      0.244986       1.87675      0.215940      0.235627    -0.0672783     -0.100694    0.00538176      0.861154       Opttag_D
      92       74.8556       2.75663       1.00000  0.000000E+00       1.00000  0.000000E+00      0.833436       38.8721       1.00000       47.7602  0.000000E+00       4.61223  0.000000E+00       4.61223  0.000000E+00  0.000000E+00      -9.22446       71.3295       2.03201        1       Scatag_D
      92       813.254       4041.31  0.000000E+00  0.000000E+00       2147.04  0.000000E+00       1921.84       2658.61  0.000000E+00       8359.72       34.4562       12491.0 -1.000000E+10 -1.000000E+10 -1.000000E+10 -1.000000E+10 -1.000000E+10  0.000000E+00  0.000000E+00  0.000000E+00  0.000000E+00  0.000000E+00  0.000000E+00  0.000000E+00  0.000000E+00  0.000000E+00  0.000000E+00  0.000000E+00  0.000000E+00       Funtag_D
      93  1.885046E+05  1.561918E+05       32312.8       16.9648      0.246317       1.87963      0.215968      0.235832    -0.0674032     -0.101081    0.00539158      0.862446       Opttag_D
      93       74.8741       2.75898       1.00000  0.000000E+00       1.00000  0.000000E+00      0.833336       38.8511       1.00000       47.8005  0.000000E+00       4.61172  0.000000E+00       4.61172  0.000000E+00  0.000000E+00      -9.22344       71.3295       2.03201        1       Scatag_D
      93       816.225       4053.45  0.000000E+00  0.000000E+00       2145.07  0.000000E+00       1910.20       2663.50  0.000000E+00       8331.82       34.3499       12358.2 -1.000000E+10 -1.000000E+10 -1.000000E+10 -1.000000E+10 -1.000000E+10  0.000000E+00  0.000000E+00  0.000000E+00  0.000000E+00  0.000000E+00  0.000000E+00  0.000000E+00  0.000000E+00  0.000000E+00  0.000000E+00  0.000000E+00  0.000000E+00       Funtag_D
      94  1.884638E+05  1.562194E+05       32244.5       34.2738      0.248068       1.88967      0.216011      0.236073    -0.0675625     -0.101557    0.00540580      0.865669       Opttag_D
      94       74.8968       2.76170       1.00000  0.000000E+00       1.00000  0.000000E+00      0.833208       38.8227       1.00000       47.8588  0.000000E+00       4.61146  0.000000E+00       4.61146  0.000000E+00  0.000000E+00      -9.22293       71.3295       2.03201        1       Scatag_D
      94       820.535       4083.81  0.000000E+00  0.000000E+00       2143.02  0.000000E+00       1901.26       2710.17  0.000000E+00       8304.74       34.4186       12246.5 -1.000000E+10 -1.000000E+10 -1.000000E+10 -1.000000E+10 -1.000000E+10  0.000000E+00  0.000000E+00  0.000000E+00  0.000000E+00  0.000000E+00  0.000000E+00  0.000000E+00  0.000000E+00  0.000000E+00  0.000000E+00  0.000000E+00  0.000000E+00       Funtag_D
      95  1.883289E+05  1.562042E+05       32124.7       15.2199      0.247339       1.88572      0.215992      0.235982    -0.0674748     -0.101341    0.00535665      0.862209       Opttag_D
      95       74.8848       2.75944       1.00000  0.000000E+00       1.00000  0.000000E+00      0.833246       38.8274       1.00000       47.8403  0.000000E+00       4.61156  0.000000E+00       4.61156  0.000000E+00  0.000000E+00      -9.22311       71.3295       2.03201        1       Scatag_D
      95       805.682       4051.22  0.000000E+00  0.000000E+00       2143.59  0.000000E+00       1903.03       2635.89  0.000000E+00       8310.95       34.4974       12239.8 -1.000000E+10 -1.000000E+10 -1.000000E+10 -1.000000E+10 -1.000000E+10  0.000000E+00  0.000000E+00  0.000000E+00  0.000000E+00  0.000000E+00  0.000000E+00  0.000000E+00  0.000000E+00  0.000000E+00  0.000000E+00  0.000000E+00  0.000000E+00       Funtag_D
      96  1.882566E+05  1.562062E+05       32050.4       9.26619      0.247641       1.88770      0.215997      0.236022    -0.0674863     -0.101416    0.00532864      0.861527       Opttag_D
      96       74.8873       2.75930       1.00000  0.000000E+00       1.00000  0.000000E+00      0.833223       38.8195       1.00000       47.8516  0.000000E+00       4.61146  0.000000E+00       4.61146  0.000000E+00  0.000000E+00      -9.22293       71.3295       2.03201        1       Scatag_D
      96       797.280       4044.81  0.000000E+00  0.000000E+00       2142.92  0.000000E+00       1902.07       2614.87  0.000000E+00       8304.53       34.5828       12209.3 -1.000000E+10 -1.000000E+10 -1.000000E+10 -1.000000E+10 -1.000000E+10  0.000000E+00  0.000000E+00  0.000000E+00  0.000000E+00  0.000000E+00  0.000000E+00  0.000000E+00  0.000000E+00  0.000000E+00  0.000000E+00  0.000000E+00  0.000000E+00       Funtag_D
      97  1.882091E+05  1.562068E+05       32002.3       9.91142      0.248023       1.89026      0.216003      0.236066    -0.0674896     -0.101493    0.00530918      0.860884       Opttag_D
      97       74.8906       2.75888       1.00000  0.000000E+00       1.00000  0.000000E+00      0.833187       38.8094       1.00000       47.8681  0.000000E+00       4.61144  0.000000E+00       4.61144  0.000000E+00  0.000000E+00      -9.22288       71.3295       2.03201        1       Scatag_D
      97       791.466       4038.78  0.000000E+00  0.000000E+00       2142.02  0.000000E+00       1901.44       2614.52  0.000000E+00       8296.58       34.6680       12182.8 -1.000000E+10 -1.000000E+10 -1.000000E+10 -1.000000E+10 -1.000000E+10  0.000000E+00  0.000000E+00  0.000000E+00  0.000000E+00  0.000000E+00  0.000000E+00  0.000000E+00  0.000000E+00  0.000000E+00  0.000000E+00  0.000000E+00  0.000000E+00       Funtag_D
      98  1.881632E+05  1.561996E+05       31963.6       20.5290      0.248346       1.89305      0.216000      0.236110    -0.0674481     -0.101531    0.00529673      0.859150       Opttag_D
      98       74.8904       2.75716       1.00000  0.000000E+00       1.00000  0.000000E+00      0.833136       38.7972       1.00000       47.8896  0.000000E+00       4.61137  0.000000E+00       4.61137  0.000000E+00  0.000000E+00      -9.22275       71.3295       2.03201        1       Scatag_D
      98       787.758       4022.53  0.000000E+00  0.000000E+00       2140.32  0.000000E+00       1901.50       2634.25  0.000000E+00       8285.37       34.4827       12157.4 -1.000000E+10 -1.000000E+10 -1.000000E+10 -1.000000E+10 -1.000000E+10  0.000000E+00  0.000000E+00  0.000000E+00  0.000000E+00  0.000000E+00  0.000000E+00  0.000000E+00  0.000000E+00  0.000000E+00  0.000000E+00  0.000000E+00  0.000000E+00       Funtag_D
      99  1.881060E+05  1.561977E+05       31908.3       8.39653      0.248722       1.89573      0.216000      0.236159    -0.0674370     -0.101588    0.00529274      0.858368       Opttag_D
      99       74.8921       2.75631       1.00000  0.000000E+00       1.00000  0.000000E+00      0.833091       38.7865       1.00000       47.9076  0.000000E+00       4.61122  0.000000E+00       4.61122  0.000000E+00  0.000000E+00      -9.22243       71.3295       2.03201        1       Scatag_D
      99       786.573       4015.21  0.000000E+00  0.000000E+00       2139.09  0.000000E+00       1900.88       2603.23  0.000000E+00       8276.89       34.3311       12152.1 -1.000000E+10 -1.000000E+10 -1.000000E+10 -1.000000E+10 -1.000000E+10  0.000000E+00  0.000000E+00  0.000000E+00  0.000000E+00  0.000000E+00  0.000000E+00  0.000000E+00  0.000000E+00  0.000000E+00  0.000000E+00  0.000000E+00  0.000000E+00       Funtag_D

   MaxLik Scaling Cycle 0104

   Wilson plot

   Statistics in resolution bins after refinement cycle 0003
```


|  |  |
| --- | --- |
|  | - Rxpct factors plot- Rxpct factors table- <Log-Lik Gain>- Ln Sigma A- Correlation Coefficients |


```
   Iters     Tot_Funct    Xray_Funct    Geom_Funct          Grms     rmsD2init     maxD2init             R         Rfree           LLG       LLGfree     rms(bond)    rms(angle)
   Iters             K   B_IMPF_FRAG        K_MISS   B_TEMP_MISS   K_IMPF_MISS   B_IMPF_MISS        K_SOLV   B_TEMP_SOLV   K_IMPF_SOLV   B_IMPF_SOLV        B_TEMP     B_TEMP_11     B_TEMP_12     B_TEMP_22     B_TEMP_13     B_TEMP_23     B_TEMP_33      K_WILSON      B_WILSON      FGCYCLES
   Iters          BOND         ANGLE      IMPROPER       TORSION    SINTORSION      TRIGONAL        PLANAR  BCORRELATION       CONTACT     IDEALDIST        CHIRAL    SIMILARITY       OCCSSUM  UTILDISTANCE     UTILANGLE   UTILTORSION        RHOFIT        ADP-01        ADP-02        ADP-03        ADP-04        ADP-05        ADP-06        ADP-07        ADP-08        ADP-09        ADP-10      RHOFITPC            QM
     100  1.880758E+05  1.561926E+05       31883.2       7.34066      0.248830       1.89685      0.215995      0.236177    -0.0674075     -0.101581    0.00529221      0.857633       Opttag_D
     100       74.8903       2.75520       1.00000  0.000000E+00       1.00000  0.000000E+00      0.833066       38.7811       1.00000       47.9156  0.000000E+00       4.61111  0.000000E+00       4.61111  0.000000E+00  0.000000E+00      -9.22222       71.3295       2.03201        1       Scatag_D
     100       786.414       4008.33  0.000000E+00  0.000000E+00       2138.21  0.000000E+00       1900.60       2598.65  0.000000E+00       8271.81       34.1370       12145.0 -1.000000E+10 -1.000000E+10 -1.000000E+10 -1.000000E+10 -1.000000E+10  0.000000E+00  0.000000E+00  0.000000E+00  0.000000E+00  0.000000E+00  0.000000E+00  0.000000E+00  0.000000E+00  0.000000E+00  0.000000E+00  0.000000E+00  0.000000E+00       Funtag_D
   Geometry Restraint Deviations SCREEN Information

 
 No -bcorrel_zeroocchydrogens_factor flag found - keeping default: will set B for OCC=0 hydrogens to    1.200 times heavy-atom B


 TLS information. Total number of groups:     2


 Group   Natoms    FixRB   FixTLS   Group Tag    Group Spec
     1     2768      Y        Y     tls1    { A|* }
     2     2770      Y        Y     tls2    { B|* }
 
NOTE BUSTER_TLS_SET tls1 { A|* }
NOTE BUSTER_TLS_T   tls1     -4.99974E-02  8.17891E-02  2.87735E-02 -4.37364E-02  3.25931E-02 -5.24938E-02
NOTE BUSTER_TLS_L   tls1      1.34630E+00  5.19551E-01  5.36731E-01 -3.76866E-01 -6.37599E-01  1.69109E-01
NOTE BUSTER_TLS_S   tls1      1.07403E-01 -2.67331E-01  3.34085E-01 -2.69031E-01  1.27949E-01 -7.05510E-02  3.27382E-02 -1.30945E-01
NOTE BUSTER_TLS_O   tls1     -2.32263E+01 -8.41675E+01  1.09752E+01
NOTE BUSTER_TLS_FIX tls1 ALL
 
NOTE BUSTER_TLS_SET tls2 { B|* }
NOTE BUSTER_TLS_T   tls2     -6.98594E-02 -1.86037E-02  3.61751E-02  9.32305E-03  3.30742E-02 -3.60360E-03
NOTE BUSTER_TLS_L   tls2      6.97425E-01  3.84731E-01  4.14949E-01  2.96503E-01 -4.03999E-01 -3.04761E-01
NOTE BUSTER_TLS_S   tls2      8.24345E-03  4.73999E-02 -7.39498E-02  8.97429E-02  1.02197E-01  9.18372E-03 -3.17101E-02  3.38103E-02
NOTE BUSTER_TLS_O   tls2      2.22079E+01 -7.23190E+01  3.56420E+00
NOTE BUSTER_TLS_FIX tls2 ALL

 For final position, Geometry function summary
     term                           number     weight       rms        GooF      function     fn/numb
     ================================================================================================
     BOND bond lengths (angs)       5653       2.000      0.0053       0.264       786.41       0.139
     ANGL bond angles (degs)        7670       2.000        0.86       0.511      4008.33       0.523
     TORS split-harmonic tors       1270       0.000       16.84       1.059         0.00       0.000
     SINTOR sinusoidal tors         1270       2.000       16.84                  2138.21       1.684
     PLAN general plane (angs)       963       5.000      0.0126       0.628      1900.60       1.974
     BCORrelations (angs^2)         5653      10.000       0.601       0.214      2598.65       0.460
     CONT (bad contacts)               0       5.000                                 0.00
     IDEAL(ideal-dist contact)      5123       4.000                              8271.81
     CHIRAL (gelly semiharmon)       740       5.000                                34.14
     SIM similarity restraint for NCS and/or TARGET                              12145.03
     OCCSUM restraint on sum of occupanies                                           0.00       as none defined
     DISTAN utility restraints interatomic distance                                  0.00       as none defined
     UTILANGLE utility angle restraints                                              0.00       as none defined
     UTILTOR utility restraints on torsion angles                                    0.00       as none defined
     Peptide omega torsion angles                           2.68 degs - monitor only

 Weighted rms      0.0053         0.858  (temporary rms bond and angle for BUSTER to pick up!)
 Weighted intra-residual rms(bond) for StdProtein (excluding H)      0.0051
 Weighted intra-residual rms(bond) for Buffer     (excluding H)      0.0027
 Outputing geometry progress graph in plotmtv format to file:Geom_Res.mtv

 ############### Running final geometry/screen analysis (screen_final.txt)

 s/r gelly normal termination
```

Statistics and scale factors vs. cycle number
```

|  |  |  |
| --- | --- | --- |
|  | - Rxpct factors- <Log-Lik Gain>- GEOM Residuals- Ln Sigma_A_ | - Scale Factors- B Factors- Imperfection Factors |

```
   Final Geometry Information


---


### 2. Final results

Statistics and scale factors vs. cycle number
```

|  |  |  |
| --- | --- | --- |
|  | - Rxpct factors- <Log-Lik Gain>- GEOM Residuals- Ln Sigma_A_ | - Scale Factors- B Factors- Imperfection Factors |

```
   Average Figure of Merit
   Structure Factor Amplitudes


 # normal termination

  Program stopped.
```

closesok message: time spent in tree = 1847.08 (user) 38.60 (sys)

---

# Normal termination

```
 Resources usage summary:
     Time spent in client ...................  1847.09 (user)       38.63 (system)
     Time spent in scripts (    27 calls) ...     3.47 (user)        0.20 (system)
     Time spent in server ...................     0.00 (user)        0.01 (system)

     Max resident memory used (kb) .......... 467708
     Number of page faults .................. 0 (major)    569907 (minor)
     Number of context switches ............. 1676296 (niced)    7974 (other)
```
